# Life course socioeconomic position and cognitive aging in later life: A scoping review

**DOI:** 10.1016/j.alcr.2025.100670

**Published:** 2025-03-05

**Authors:** Mengling Cheng, Lore Van Herreweghe, Aswathikutty Gireesh, Stefan Sieber, Kenneth F. Ferraro, Stéphane Cullati

**Affiliations:** a School of Social and Public Administration, East China University of Science and Technology, China; b Swiss Centre of Expertise in Life Course Research, University of Lausanne, Switzerland; c Centre for Sociological Research, KU Leuven, Belgium; d Department of Behavioural Science and Health, Institute of Epidemiology and Health Care, University College London, United Kingdom; e Barcelona Institute for Global Health (ISGlobal), Spain; f Center on Aging and the Life Course, Purdue University, United States; g Department of Sociology, Purdue University, United States; h Population Health Laboratory (#PopHealthLab), University of Fribourg, Switzerland

**Keywords:** Cognition, Aging, Socioeconomic position, Life course, Social determinants of health

## Abstract

**Background and Objectives:**

Low socioeconomic position (SEP) throughout the life course is related to poorer cognitive health in later life, but debate ensues on the life course models for this association. To advance inquiry on the topic, we conducted a scoping review.

**Research Design and Methods:**

We examined the association between life course SEP and cognitive function in later life in observational studies—considering cognition both as a cross-sectional level and as a longitudinal trajectory across cognitive domains—and assessed whether the empirical evidence supported life course models. We focused on studies in the general population with cognition measured in the second half of life (45 +). Forty-two studies (21 datasets) were included representing 595,276 participants (201,375 across unique datasets) from 46 countries.

**Results:**

For cognitive level, studies consistently found associations between SEP at various stages of the life course, both in overall cognition and across specific cognitive domains. These associations were generally robust to confounding and mediating factors. For cognitive trajectory, studies showed inconclusive associations with SEP across life course and across cognitive domains. Results supported the sensitive period, pathway, and accumulation models, but not the critical period model. Results supported that education acts as a pathway (and potential mediator) in the association between early-life SEP and later-life cognition.

**Discussion and Implications:**

SEP throughout the life course has a robust association with later-life cognitive level, but not decline. Early-life cognitive enrichment for young people raised in socioeconomically disadvantaged households may reduce the SEP gap in cognitive functioning during later life.

## 1. Background and objectives

With an increasingly older population and a concomitant increase in the health and economic impact of premature cognitive decline, it is important to understand modifiable risk factors associated with cognitive impairment in later life, especially social and environmental factors (Chen et al., 2022; Kramer et al., 2004; Xu et al., 2016; Zhao et al., 2021). Life course studies offer the unique opportunity to study the risk factors for cognitive impairment across life stages and the mechanisms that underpin them (Kuh et al., 2003). They also help in identifying how social stratification processes throughout an individual’s life shape cognitive health in later life (Liu et al., 2010; Power et al., 2013; Tucker-Drob, 2019). These life course studies often delineate four, partly overlapping, life course models: critical period, sensitive period, pathway, and accumulation model (Ben-Shlomo et al., 2016). These models have rarely been evaluated with empirical evidence in cognitive aging studies.

Cognitive function is an umbrella term comprising both fluid and crystallised cognitive abilities and can be further divided into different cognitive domains (Tucker-Drob, 2019). Variations in these cognitive domains are an integral part of aging, with some domains more likely to be affected by aging than others (Harada et al., 2013). Yet, individual differences in various cognitive domains are correlated (Tucker-Drob, 2009), suggesting that individuals experiencing weaknesses in one domain will tend to have weaknesses in other domains as well (Harvey, 2019; Tucker-Drob, 2019). Any decline in cognitive function and specific cognitive domains often leads to loss of independence and quality of life, and a higher risk of premature mortality (Deary et al., 2009; Livingston et al., 2020).

Past research has suggested that socioeconomic position (SEP) could influence the baseline cognitive function – referring to the initial measurement of an individual’s cognition at the start of a study – and changes in cognitive levels, or both (Kaplan et al., 2001). SEP is defined as a social condition structured by factors that enable differential access of individuals to material, social and cultural resources and opportunities (Bartley, 2016; Bourdieu, 1986; Weber, 1946) and can be operationalized through four key dimensions: education, income, occupation, and wealth (Hällsten & Thaning, 2022). Socioeconomic disadvantage is related to distinct cognitive domains through two mechanisms (Ursache & Noble, 2016): first, SEP shapes the linguistic and intellectual environment in which individuals live, thereby stimulating the language cortex and its associated language cognitive domain; second, SEP shapes the exposure of individuals to environmental and psychosocial stressors, which could in turn influence the hippocampus, amygdala and prefrontal cortex, which influence memory, social-emotional processing and self-control. These two mechanisms align with the behaviour-cultural and inter-personal types of social exposures described in Bartley (2016) and can be articulated with biological processes (material, central nervous system-mediated, epigenetic) to explain how social factors can become biological (Blane et al., 2013). How SEP throughout the life course is related to cognitive health across cognitive domains in later life is not well understood. Also, whether the life course models are supported by empirical evidence remains unclear. However, a few studies have explicitly engaged with life course models: Lee and Schafer (2021), Künzi et al. (2021), Greenfield et al. (2020), Ding and He (2021), Faul et al. (2021), Ye et al. (2022), and Ford et al. (2022) tested the pathway model by examining the mediating role of adulthood SEP between childhood SEP and later-life cognition. Aartsen et al. (2019) and Cheval et al. (2019) interpreted their findings in the context of life course models, such as the latency, pathway, and cumulative models. Luo and Waite (2005) provided evidence supporting the accumulation model, showing that SEP across life course periods influences later-life cognition.

In cognitive aging studies, however, it is common to find studies examining the influence of SEP indicators across the life course (e.g., childhood SEP) without explicitly engaging with a life course theoretical lens or testing specific life course models. While these studies do not formally evaluate such models using dedicated statistical approaches, their findings can indirectly support one or more life course models. To better understand the extent of empirical support for life course models, a review is needed that considers both studies explicitly testing these models and those providing indirect evidence through findings relevant to life course theories.

This scoping review differs from existing ones (Yaple & Yu, 2019; Valenzuela & Sachdev, 2006) by adopting a life course perspective and consolidating evidence across multiple SEP dimensions, cognitive outcomes, and life course frameworks. Given the variability in cognitive outcomes, particularly across cognitive domains, this review does not aim to test specific hypotheses about the influence of life course SEP on particular cognitive domains. Instead, it seeks to synthesize and map the evidence, providing an overview of the breadth and patterns of findings. The goal of this scoping review is to map the relationship of life course SEP with levels of cognitive function and rates of cognitive decline in later life across cognitive domains. Furthermore, this review aims to assess the extent to which empirical evidence supports life course models, providing insights into how SEP influences cognitive aging through different mechanisms and at various life stages.

## 2. Research design and methods

### 2.1. Definition of life course models

We assessed four life course models: critical period, sensitive period, pathway and accumulation.

The “critical period” model states that some exposures, when occurring in a specific time window of early development, will cause irreversible damage to later health (Blane et al., 2007). The critical period is an extension of the biological or foetal programming hypothesis to explain the long-lasting health effects of experiences in early uterine life, regardless of subsequent exposures to protective or risk factors (Kwon & Kim, 2017; Nelson & Gabard-Durnam, 2020). A familiar example is the well-established association between intrauterine nutritional deprivation and increased risk of cardiovascular diseases in the progeny (Barker et al., 1993). Despite criticism of the choice of the term “critical” (Bailey et al., 2001; Skogen & Øverland, 2012) and the lack of consolidated empirical support from previous studies (Wagner et al., 2022), the critical period model continues to be considered in research that synthesises evidence (Power et al., 2013).

The “sensitive period” model states that an exposure during a specific developmental phase of life (e.g., childhood, adolescence) may have enduring health consequences (Mishra et al., 2010). Contrary to the critical period, the sensitive period model hypothesises there is some scope to modify or even reverse those consequences outside that specific period (Ben-Shlomo & Kuh, 2002), in particular thanks to accumulated reserves (Cullati et al., 2018). The effect of the sensitive period can be illustrated with the English and Romanian Adoptees study, which demonstrated deep-seated effects of early childhood nutritional deprivation in the cognitive development of children (Mehta et al., 2009). An extended analysis using the same data showed that the effects persist even into adulthood, but in an attenuated way: the deprived Romanian adoptees had more alterations in brain structure compared with the non-deprived UK adoptees, despite having access to many enriching resources (Mackes et al., 2020).

The “pathway” model, or “chain of risks” model, recognises the impact of later-life modifiers and posits that exposures (e.g., poor socioeconomic circumstances) early in life are associated with poor health outcomes later in life (Whalley et al., 2006) through a series of sequential risks (Singh-Manoux et al., 2004). The pathway model describes a sequence of exposures that are chronologically linked in time. For example, a recent study by Lee and Schafer (2021) observed that the association between positive childhood experiences and adult cognition operates through education and psychosocial resources.

The “accumulation” model proposes that socioeconomic adversities or disadvantages accumulate over lifetime, leading to poor health outcomes in later life (Cable, 2014) and can be transmitted across generations (Ferraro & Shippee, 2009). Socioeconomic disadvantages are generally thought in terms of objective conditions and their duration, which structure their exposure to risks and opportunities over their life course (Elder et al., 2003). In addition to objective conditions, the accumulation model also considers individuals’ perceptions of disadvantage as an agency factor, which could mitigate objective conditions (Ferraro et al., 2009), notably the way in which individuals are imbued with a sense of their position relative to others (Bourdieu, 1989). Earlier studies have documented that cumulative disadvantage over the life course is associated with multimorbidity (Jungo et al., 2022) and mortality (Demakakos et al., 2016; Johnson-Lawrence et al., 2015; Killewald et al., 2017). Some studies reported a similar link between accumulated socioeconomic disadvantage and poor cognitive function in later life, although the evidence is not conclusive (Haan et al., 2011; Hazzouri, 2011).

### 2.2. Search strategy

We conducted a scoping review without a systematic literature search. We followed the Preferred Reporting Items for Systematic reviews and Meta-Analyses extension for Scoping Reviews (PRISMA-ScR) throughout the review process (Tricco et al., 2018). The PRISMA-ScR Checklist can be found in the Supplementary Material.

**Fig. 1.**
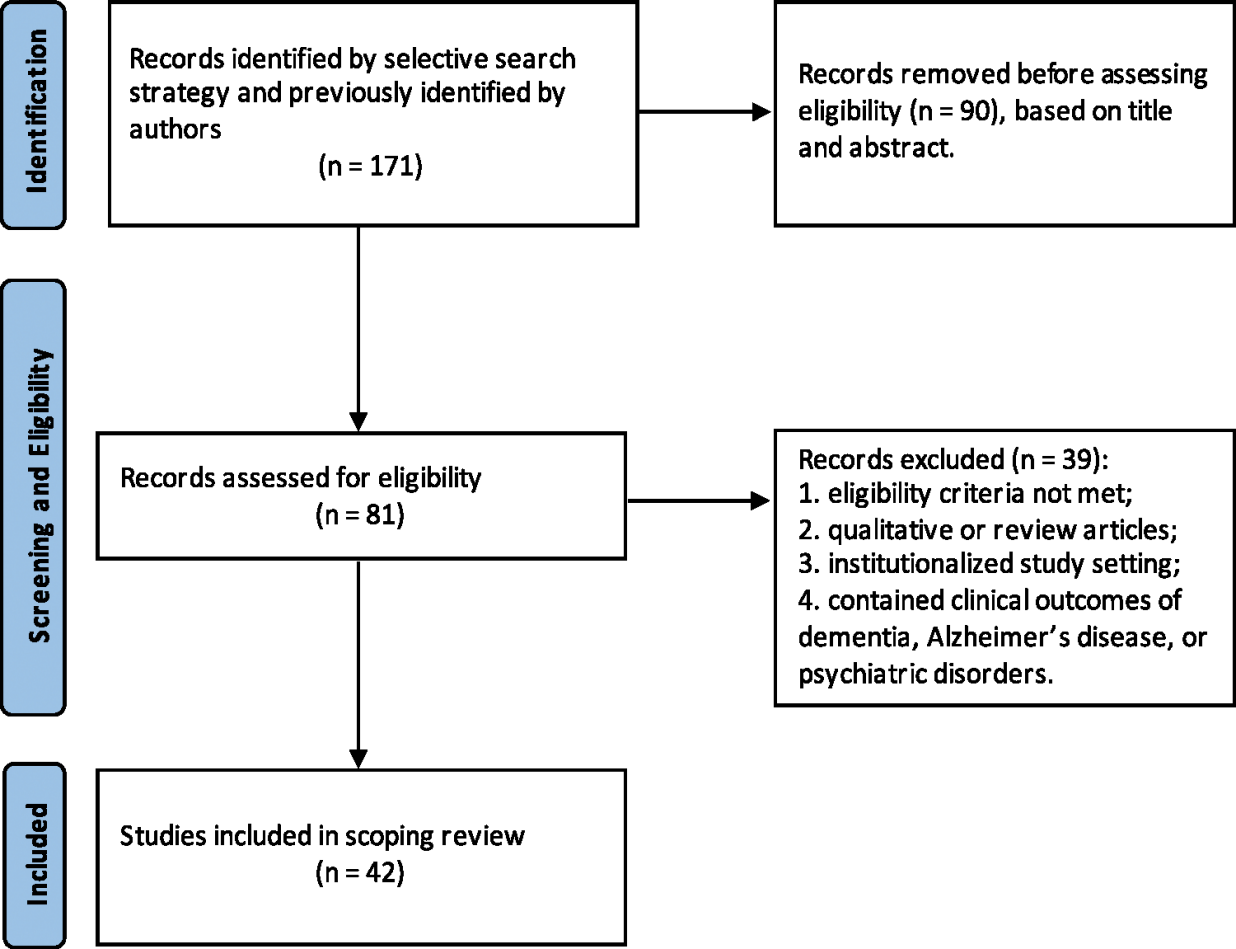
PRISMA flow diagram.

We identified articles through non-systematic but widely used approaches: including studies recommended by experts who are familiar with this research area, screening reference lists of included studies for additional relevant articles with Google Scholar, scanning the “similar articles” and “cited by” sections of included studies in PubMed, and manually searching key journals (Fig. 1).

### 2.3. Eligibility criteria

Included studies adhered to the subsequent criteria: 1) studied general populations; 2) measured/observed cognition in the “second half of life” (i.e., at least 45 years old); 3) reported any cognitive measures assessed with cognitive tests, whose data were combined in scores (observed or latent) or analysed separately, such as memory (e.g., immediate recall, delayed recall, etc.), language or verbal skills (e.g., fluency, reading and comprehension, etc.), executive functioning (e.g., reasoning, problem solving, etc.), attention and concentration (e.g., selective attention, sustained attention/vigilance, etc.), processing speed (e.g., coding and tracking, etc.); 4) one or more other SEP indicators at the individual or household level (e.g., education, income, wealth, occupation, employment status, household conditions, number of books at home), at the meso-level (e.g., economic or socioeconomic information aggregated at the school, neighbourhood, or community level), or at the macro-level (e.g., economic or socioeconomic information aggregated at the national level or above); 5) measured SEP indicators either before (childhood or adolescence, young adulthood, middle age) or concurrent (older age) to the cognitive outcome, and preferences were given to the former case (i.e., before). For this study, SEP was broadly defined as an umbrella term for the social and economic factors that influence the position of individuals in their society (Shaw et al., 2007).

Studies were excluded if they did not meet the above-mentioned criteria, if they were qualitative research or review articles, if they were conducted among institutionalized populations, and if they contained clinical outcomes of dementia, Alzheimer’s disease, or psychiatric disorders. Excluded studies are listed in the Supplementary Material (Table S1). Additionally, some relatively recent studies, or those identified late in the literature search, were excluded because they did not meet at least one of the following criteria: they did not (1) provide new key characteristics that would enrich the scope of this review, such as novel datasets, additional countries, new SEP indicators, or explicit tests of life course models; or (2) demonstrate mediation effects between life course SEP indicators.

### 2.4. Data extraction

We extracted data on article characteristics, measures of cognition and cognitive domains, measures of life course SEP, covariates in the final model, results of the association between SEP and cognitive levels and/or declines, interpretation of results by theoretical frameworks, limitations, and whether sensitivity analyses were included.

### 2.5. Data synthesis

We provide an overview of each study and the cognitive domains that were studied (Table 1); table entries are listed chronologically by year of publication. Using the same order, we also summarized the results of associations between life course SEP and cognitive levels and/or decline (Table 2). In addition, we summarized the SEP indicators used in each study by life course periods (Table S2). We also summarized the results of associations with later-life global cognition (Table 3) and cognitive domains (Table S3) for each of the four dimensions of SEP (education, income, occupation and wealth; Hällsten & Thaning, 2022), at the individual-level and across life course periods.

To better synthesize the data, we classified the following five dimensions: type of cohort and age information, life course SEP periods, cognitive domains, cognitive levels or trajectories, and life course models. First, we classified the study design into five types of cohorts: birth cohort (individuals born during a given year or period), school cohort (students attending the same educational setting or programme at the same time), occupational cohort (workers from a particular profession, industry, or company), twin cohort, or older age cohort (individuals aged 45 and older). We provided the age information of the cohort. This classification was essential to contextualize the findings from the studies, understand the influence of various historical, contextual, occupational, educational, and later-life factors (such as retirement, work transitions, or health changes) on cognitive outcomes, and to assess the generalizability of the results. Second, we classified measures of SEP based on the life course periods, i.e., childhood or adolescence, young adulthood, middle age, and older age. Additionally, we examined educational level or education years separately, as education is not merely related to one specific period in life. Third, we classified measures of cognition based on the cognitive domains (Harvey, 2019): sensation and perception; motor skills and construction; attention and concentration; memory; executive functioning; processing speed; and language or verbal skills. These classifications are justified by the fact that different cognitive domains are influenced by different SEP indicators in certain life course periods, as the underlying neurophysiological mechanisms may function differently across cognitive domains, making some cognitive domains especially sensitive to specific life course SEP periods (Greenfield & Moorman, 2019). Fourth, we distinguished the results on associations between life course SEP periods and (a) cognitive levels (i.e., describing mean cross-sectional levels or baseline status of cognition, e.g., latent intercepts or any overall estimates without temporality) and (b) cognitive trajectories (i.e., describing the process of intra-individual decline in cognition over aging, e.g., any estimates of slopes or rate of change). Fifth, we classified the results on associations between life course SEP periods and cognitive levels and/or trajectories by four life course models: critical period model, sensitive period model, pathway model, and accumulation model. Due to the limited number of studies that disaggregated cognitive outcomes by gender, we were unable to examine and compare how inequalities in cognitive outcomes may vary between women and men.

This appraisal was inspired by the life course causal models of Wagner et al. (2022). The critical period model is supported if a study shows that childhood or adolescent SEP alone is associated with later-life cognitive outcomes (i.e., no SEP indicators in any other period are related to later-life cognition) and was not attenuated after adjusting for later-life SEP indicators. The sensitive period model is supported if a study shows that SEP indicators in any life course periods, particularly in the developmental period of childhood or adolescence, are associated with later-life cognitive outcomes, and if the association was attenuated but remained significant after adjustment for later-life SEP indicators. The pathway model is supported if a study shows that the effects of SEP indicators in childhood or adolescence or education are mediated by SEP indicators in later life course periods (i.e., young adulthood, middle age, or older age), either through partial mediation or full mediation. The accumulation model is supported if a study shows that SEP indicators in a minimum of two life course periods (i.e., childhood & adolescence, education, young adulthood, middle age, or older age), or an operationalisation of the SEP indicators over the life course to measure some form of accumulation (i.e., cumulative SEP), are associated with later-life cognitive levels/trajectories.

In addition, the geographic location or society (place) and the historical period (time) in which individuals live may have an impact on their cognition (Elder, 1994). The principle of “place and time” is one of the five fundamental principles of life course research (Elder & Shanahan, 2006), indicating that every individual’s life course is embedded within and shaped by specific historical and spatial contexts. To account for this in this scoping review, we identified studies explicitly considering the aspect of “place and time” in the associations between life course SEP and cognitive levels, as well as cognitive trajectories.

## 3. Results

### 3.1. Study characteristics

The 42 studies included 21 datasets covering 37 high-income countries, six upper-middle-income countries, and three lower-middle-income countries (Table 1). Further study characteristics are reported in the Supplementary Material (**Supplementary Text 1**). The included studies used prospective datasets (n = 23) or ambidirectional datasets (n = 19), which combined retrospective and prospective information. The total number of participants across all 42 studies was 595,276 (201,375 across unique datasets). Participants were 45 years or older at the first measurement of cognitive function. The age range of participants varied from 45 to 105 years.

Six cognitive domains were studied, and the most studied domain was memory (n = 24), followed by language/verbal skills (n = 10), executive functioning (n = 8), processing speed (n = 4), sensation/perception (n = 2) and motor skills (n = 1). A global cognition score was studied in 23 studies.

All studies included a measure of education and a measure of SEP (e.g., income, family financial status) during at least one of the following four life course periods: childhood or adolescence, young adulthood, middle age, and older age (Table S2). In majority of the studies, childhood or adolescent SEP was measured by parental educational attainment (Brewster et al., 2014; Brown, 2010; Chen et al., 2022; Ding & He, 2021; Everson-Rose et al., 2003; González et al., 2013; Greenfield et al., 2020; Jefferson et al., 2011; Künzi et al., 2021; Lee & Schafer, 2021; Luo & Waite, 2005; Lyu & Burr, 2016; Marden et al., 2017; Peterson et al., 2021; Reynolds et al., 2022; Selvamani & Arokiasamy, 2021; Wilson et al., 2005; Yang & Wang, 2020; Ye et al., 2022; Zeng et al., 2022; Zhao et al., 2021; Zhou et al., 2022) and father’s principal occupation (Aartsen et al., 2019; Brown, 2010; Cheval et al., 2019; Ericsson et al., 2017; Everson-Rose et al., 2003; Faul et al., 2021; Ford et al., 2022; Fritze et al., 2014; Greenfield et al., 2020; Hurst et al., 2013; Jefferson et al., 2011; Künzi et al., 2021; Landy et al., 2017; Leist et al., 2021; Luo & Waite, 2005; Marden et al., 2017; Moorman et al., 2019; Ritchie et al., 2016; Selvamani & Arokiasamy, 2021; Staff et al., 2016; Wilson et al., 2005; Yang & Wang, 2020; Zaninotto et al., 2018; Zeng et al., 2022; Zhou et al., 2022) during the respondent’s childhood or adolescence. Other indicators of childhood or adolescent SEP include housing quality (Aartsen et al., 2019; Cheval et al., 2019), family income or financial situation during this (early) life period (Cermakova et al., 2018; Everson-Rose et al., 2003; Faul et al., 2021; Luo & Waite, 2005; Lyu & Burr, 2016; Marden et al., 2017; Moorman et al., 2019; Peterson et al., 2021; Wilson et al., 2005) as well as measures of overcrowding (Aartsen et al., 2019; Cermakova et al., 2018; Wolfova et al., 2021).

For indicators pointing to periods after childhood, we see that in contrast with childhood or adolescent SEP, not every study includes such measurements of SEP later in life. Some studies solely include childhood SEP in their analyses without any measure of SEP later in life (Barnes et al., 2012; Brewster et al., 2014; Ericsson et al., 2017; Everson-Rose et al., 2003; Yang & Wang, 2020). Exceptions are studies that included a specific measure of SEP in young adulthood, either first occupation of the respondent (Künzi et al., 2021; Leist et al., 2014; Zeng et al., 2022) or a standardized test score during high school (Greenfield et al., 2020).

All studies included both childhood or adolescent SEP and education, and examined them either alone (Barnes et al., 2012; Brewster et al., 2014; Chen, 2016; Ericsson et al., 2017; Everson-Rose et al., 2003; Reynolds et al., 2022; Yang & Wang, 2020) or in combination with other life course SEP.

For middle-age, we count 13 studies that include measures of SEP during this period. Majority of studies include the respondent’s principal occupation position (Aartsen et al., 2019; Cheval et al., 2019; Faul et al., 2021; Ford et al., 2022; Greenfield et al., 2020; Hurst et al., 2013; Jefferson et al., 2011; Künzi et al., 2021; Landy et al., 2017; Moorman et al., 2019; Ritchie et al., 2016; Selvamani & Arokiasamy, 2021; Staff et al., 2016; Wahrendorf et al., 2020; Wilson et al., 2005; Zhang et al., 2008) or total household income (Greenfield et al., 2020; Jefferson et al., 2011; Moorman et al., 2019; Zeng et al., 2022) before retirement.

Finally, measures of older-age SEP are included in 14 of our selected studies, and most of the time using household income (Brown, 2010; Ding & He, 2021; Jefferson et al., 2011; Künzi et al., 2021; Leist et al., 2014; Luo & Waite, 2005; Maharani, 2020; Wolfova et al., 2021; Ye et al., 2022; Zhou et al., 2022), satisfaction with household income (Aartsen et al., 2019; Cheval et al., 2019) or wealth (Marden et al., 2017; Selvamani & Arokiasamy, 2021; Zaninotto et al., 2018).

In sum, childhood or adolescent SEP and education are the most frequently used indicators of life course SEP in our selected studies. However, even within these studies, there is a wide variety in the measures used. It is also evident that young adulthood is a largely understudied period when assessing the relationship between life course SEP and late-life cognition. Finally, most of the SEP indicators were operationalised at the individual or household level, but four studies included meso-level indicators (Jefferson et al., 2011; Leist et al., 2021) and macro-level indicators (Leist et al., 2021; Chen, 2016) in addition to individual-level SEP indicators.

### 3.2. Associations between life course SEP and cognitive levels

Table 2 (column 2) shows that all of the 42 studies found a consistent positive association between SEP and levels of cognitive function in later life across life course periods (i.e., childhood or adolescence, young adulthood, middle age, and older age) and cognitive domains.

Thirty-four studies found that higher childhood or adolescent SEP and education were related to higher levels of cognitive function in later life. Of these, twenty-six studies reported an independent effect of childhood or adolescent SEP after adjusting for SEP later in life (young adulthood, middle age, or older age). Eight studies reported an effect of childhood or adolescent SEP without adjusting for SEP later in life (Barnes et al., 2012; Brewster et al., 2014; Chen, 2016; Ericsson et al., 2017; Everson-Rose et al., 2003; Fritze et al., 2014; Reynolds et al., 2022; Yang & Wang, 2020). Among the studies that adjusted for SEP later in life, 12 additionally adjusted for childhood health conditions and 17 additionally adjusted for later-life chronic conditions — the observed association remained significant.

The association between childhood or adolescent SEP and levels of cognitive function was mediated by adulthood SEP in seven studies or was attenuated by education in 10 studies. The effect of childhood SEP also was often attenuated by education (Brown, 2010; Cermakova et al., 2018; Ding & He, 2021; Everson-Rose et al., 2003; Ford et al., 2022; Greenfield et al., 2020; Lee & Schafer, 2021; Maharani, 2020; Wilson et al., 2005; Wolfova et al., 2021), but remained associated with cognition. There are only four studies where no significant relationship between childhood SEP and level of later-life cognition was found, or where the relation was fully explained by other covariates (Greenfield et al., 2020; Künzi et al., 2021; Ritchie et al., 2016; Staff et al., 2016). We cannot draw conclusions on the study by Wahrendorf et al. (2020), as they did not report estimates of childhood SEP and only included this factor as a control in their sensitivity analyses. Three studies included indicators of macroeconomic conditions (economic recessions) at the time of birth (Chen, 2016; Fritze et al., 2014) and in middle age (Leist et al., 2014). Being exposed to economic recessions at the time of birth in Taiwan was related to lower later-life cognition; similarly, being exposed at ages 45–49 among men and 25–44 among women living in Europe was associated with lower later-life cognition.

Ten studies found that higher middle-age SEP was related to higher levels of cognitive function in later life (Ford et al., 2022; Greenfield et al., 2020; Hurst et al., 2013; Künzi et al., 2021; Landy et al., 2017; Lee & Schafer, 2021; Selvamani & Arokiasamy, 2021; Wahrendorf et al., 2020; Wilson et al., 2005; Zeng et al., 2022). The association between middle-aged SEP and levels of cognitive function was attenuated by childhood or adolescent SEP in three studies (Aartsen et al., 2019; Ding & He, 2021; Staff et al., 2016).

Fifteen studies found that higher SEP during older ages was related to higher levels of cognitive function in later life (Aartsen et al., 2019; Cheval et al., 2019; Ding & He, 2021; González et al., 2013; Jefferson et al., 2011; Leist et al., 2021; Luo & Waite, 2005; Maharani, 2020; Marden et al., 2017; Muhammad et al., 2022; Peterson et al., 2021; Selvamani & Arokiasamy, 2021; Ye et al., 2022; Zaninotto et al., 2018; Zhou et al., 2022). Exceptions were null associations between older-age SEP and memory (Künzi et al., 2021) or cognitive scores (Ritchie et al., 2016). The estimates for older-age SEP were not reported in two studies (Brown, 2010; Wolfova et al., 2021).

Twenty-three studies included a score of global cognition. The remaining 19 studies that investigated life course SEP and various cognitive domains showed consistent results across cognitive domains, except for four studies (Brewster et al., 2014; Jefferson et al., 2011; Ritchie et al., 2016; Wolfova et al., 2021). Moreover, these studies did not point to one specific cognitive domain or measure of life course SEP.

### 3.3. Associations between life course SEP and cognitive trajectories

Table 2 (column 3) shows that apart from the 19 studies which did not test cognitive decline, the evidence on association between life course SEP and trajectories of cognitive decline was inconsistent: 15 studies found that higher life course SEP was related to slower cognitive decline in later life (Aartsen et al., 2019; Barnes et al., 2012; Brewster et al., 2014; Cheval et al., 2019; Leist et al., 2021; Lyu & Burr, 2016; Marden et al., 2017; Moorman et al., 2019; Reynolds et al., 2022; Wolfova et al., 2021; Yang & Wang, 2020; Ye et al., 2022; Zaninotto et al., 2018; Zeng et al., 2022; Zhang et al., 2008), whereas eight studies (Cermakova et al., 2018; Cheval et al., 2019; Ericsson et al., 2017; Everson-Rose et al., 2003; González et al., 2013; Ritchie et al., 2016; Staff et al., 2016; Wilson et al., 2005) did not find such an association.

Twelve studies found that higher childhood or adolescent SEP was related to slower cognitive decline in later life (Aartsen et al., 2019; Brewster et al., 2014; Ericsson et al., 2017; Leist et al., 2021; Lyu & Burr, 2016; Marden et al., 2017; Reynolds et al., 2022; Wolfova et al., 2021; Ye et al., 2022; Zaninotto et al., 2018; Zeng et al., 2022; Zhang et al., 2008). Five studies found that higher education was related to slower cognitive decline in later life (Cheval et al., 2019; Faul et al., 2021; Lyu & Burr, 2016; Marden et al., 2017; Zaninotto et al., 2018). Five studies reported an independent effect of childhood or adolescent SEP after adjusting for SEP in young adulthood, middle age or older age (Aartsen et al., 2019; Marden et al., 2017; Reynolds et al., 2022; Zaninotto et al., 2018; Zhang et al., 2008), while seven studies reported an effect of childhood or adolescent SEP without adjusting for SEP later in life.

In addition, the association between childhood or adolescent SEP and cognitive trajectories often differed between women and men. Zhang et al. (2008) found that SEP conditions in early life were related to rate of cognitive decline, but only for women. According to Zaninotto et al. (2018), low childhood SEP relates to a slower decline of cognitive function for women only, while for men it relates to a steeper decline. The study by Cheval et al. (2019) highlighted that the effects of different dimensions of early-life SEP on cognitive function can be gender dependent. They found an accelerated decline in cognitive function for individuals living in an overcrowded household and having fewer books at home, for women and men, respectively.

The association between childhood or adolescent SEP and trajectories of cognitive function was also found to be different by cognitive domain. In the study by Ericsson et al. (2017), childhood SEP was not associated with decline in memory, processing speed or verbal skill. In the study by Aartsen et al. (2019), on the other hand, childhood SEP was associated with language/verbal skills but not with memory decline. The study by Leist et al. (2014) found contrasting effects of education on memory and language/verbal skills decline. The study by Zaninotto et al. (2018) found that mid-childhood SEP resulted in steeper declines in processing speed among men only. The study by Wolfova et al. (2021) found an association with decline in delayed memory recall but not with immediate memory recall and language/verbal skills.

Three studies found that higher middle-age SEP was related to slower cognitive decline in later life (Aartsen et al., 2019; Cheval et al., 2019; Moorman et al., 2019). Moorman et al. (2019) found that higher middle-age SEP was related to slower cognitive decline in memory (but not in verbal skills). Cheval et al. (2019) found that higher middle-age SEP was related to slower cognitive decline in memory (but not in language/verbal skills) among men. Furthermore, four studies found that higher older-age SEP was related to slower cognitive decline in later life (Aartsen et al., 2019; Faul et al., 2021; Marden et al., 2017; Ye et al., 2022).

### 3.4. Summary of associations between individual-level SEP dimensions across life course periods and global cognitive levels and trajectories

Table 3 summarizes the association between individual-level socio-economic position indicators across life course periods and global cognitive levels and trajectories (n = 17). As for childhood/adolescence SEP, higher parental education (Brown, 2010; Ericsson et al., 2017; Fritze et al., 2014; González et al., 2013; Jefferson et al., 2011; Leist et al., 2014; Luo & Waite, 2005; Lyu & Burr, 2016; Maharani, 2020; Selvamani & Arokiasamy, 2021; Staff et al., 2016; Yang & Wang, 2020; Zaninotto et al., 2018), higher family income (Barnes et al., 2012; González et al., 2013; Luo & Waite, 2005; Muhammad et al., 2022; Zhang et al., 2018), and non-manual parental occupation (Brown, 2010; Ericsson et al., 2017; Jefferson et al., 2011; Luo & Waite, 2005; Selvamani & Arokiasamy, 2021; Yang & Wang, 2020; Zaninotto et al., 2018) were associated with better global cognitive levels. However, the evidence regarding the association between childhood/adolescence SEP and global cognitive trajectories was mixed: three studies found protective effects of higher parental education (Brewster et al., 2014; Lyu & Burr, 2016; Zaninotto et al., 2018), while five observed null effects (Brown, 2010; Ericsson et al., 2017; González et al., 2013; Staff et al., 2016; Yang & Wang, 2020). One study reported protective effects of higher family income (Zhang et al., 2018), while another found negative effects (Barnes et al., 2012), and a third reported null effects (González et al., 2013). Similarly, one study indicated protective effects of non-manual parental occupation (Brewster et al., 2014), while one study found negative effects (Zaninotto et al., 2018), and four reported null effects (Brown, 2010; Ericsson et al., 2017; Staff et al., 2016; Yang & Wang, 2020).

As for young adulthood SEP, nine studies reported protective effects of higher education (Barnes et al., 2012; Brown, 2010; González et al., 2013; Jefferson et al., 2011; Luo & Waite, 2005; Muhammad et al., 2022; Selvamani & Arokiasamy, 2021; Yang & Wang, 2020; Zhang et al., 2018) and one study reported protective effects of non-manual occupation (Leist et al., 2014) on global cognitive levels. However, five studies found null effects of education on global cognitive trajectories (Barnes et al., 2012; Brewster et al., 2014; Brown, 2010; González et al., 2013; Zhang et al., 2018).

As for middle-age SEP, one study reported protective effects of higher income (Jefferson et al., 2011), and three studies found protective effects of non-manual occupation (Selvamani & Arokiasamy, 2021; Staff et al., 2016; Zhang et al., 2018) on global cognitive levels. Null effects of occupation on global cognitive trajectories were observed (Staff et al., 2016; Zhang et al., 2018).

As for older-age SEP, higher income (Brown, 2010; González et al., 2013; Jefferson et al., 2011; Luo & Waite, 2005; Maharani, 2020), non-manual occupation (Jefferson et al., 2011; Maharani, 2020), and wealth (González et al., 2013; Selvamani & Arokiasamy, 2021; Zaninotto et al., 2018) were associated with better global cognitive levels. However, the evidence on the effects of income and wealth on global cognitive trajectories was predominantly null for income (Brown, 2010; González et al., 2013) and wealth (González et al., 2013; Zaninotto et al., 2018).

Table S3 summarizes the associations between individual-level socioeconomic position indicators across life course periods and cognitive levels and trajectories in various domains (n = 16). A description of these associations is reported in the Supplementary Material (**Supplementary Text 2**).

### 3.5. Appraisal of empirical support for life course models

#### 3.5.1. Critical period model

None of the studies found that childhood and adolescence acted as critical periods affecting later-life cognitive function or decline independently. That is to say, the developmental phases in life were not found to be the only time periods that affect later-life cognition. This is mainly because education and other indicators of life course SEP were independently related to later-life cognition.

#### 3.5.2. Sensitive period model

Most evidence was found for the sensitive period model. 36 studies showed that SEP during developmental phases in life had a long-term effect on later-life cognition. The majority of these studies showed positive effects of better childhood or adolescent SEP on level of cognition (Aartsen et al., 2019; Barnes et al., 2012; Brown, 2010; Cermakova et al., 2018; Chen, 2016; Cheval et al., 2019; Ding & He, 2021; Ericsson et al., 2017; Everson-Rose et al., 2003; Ford et al., 2022; Fritze et al., 2014; González et al., 2013; Greenfield et al., 2020; Hurst et al., 2013; Jefferson et al., 2011; Landy et al., 2017; Lee & Schafer, 2021; Leist et al., 2014; Luo & Waite, 2005; Lyu & Burr, 2016; Maharani, 2020; Moorman et al., 2019; Muhammad et al., 2022; Reynolds et al., 2022; Selvamani & Arokiasamy, 2021; Wilson et al., 2005; Yang & Wang, 2020; Ye et al., 2022; Zeng et al., 2022; Zhang et al., 2008; Zhou et al., 2022) and on cognitive decline (Aartsen et al., 2019; Brewster et al., 2014; Leist et al., 2021; Lyu & Burr, 2016; Marden et al., 2017; Wolfova et al., 2021; Zaninotto et al., 2018). It thus appears that better childhood or adolescent SEP had a significant effect on absolute levels of cognitive function; however, it did not protect against cognitive decline in later life (Aartsen et al., 2019). Other studies also found significant effects of middle-age SEP on later-life cognition (Aartsen et al., 2019; Cheval et al., 2019; Hurst et al., 2013; Jefferson et al., 2011; Landy et al., 2017; Moorman et al., 2019; Wilson et al., 2005; Zeng et al., 2022; Zhang et al., 2008).

#### 3.5.3. Pathway model

Six studies found that the effects of childhood SEP on later-life cognitive function were attenuated by adult SEP (Brown, 2010; Greenfield et al., 2020; Lee & Schafer, 2021; Leist et al., 2014; Zeng et al., 2022; Zhang et al., 2008). Twenty-three studies showed that education functioned as an important pathway (attenuator, mediator) through which childhood or adolescent SEP is associated with later-life cognition (Aartsen et al., 2019; Barnes et al., 2012; Cheval et al., 2019; Ding & He, 2021; Everson-Rose et al., 2003; Faul et al., 2021; Ford et al., 2022; Fritze et al., 2014; González et al., 2013; Greenfield et al., 2020; Hurst et al., 2013; Jefferson et al., 2011; Luo & Waite, 2005; Lyu & Burr, 2016; Maharani, 2020; Moorman et al., 2019; Staff et al., 2016; Wilson et al., 2005; Wolfova et al., 2021; Yang & Wang, 2020; Ye et al., 2022; Zaninotto et al., 2018; Zeng et al., 2022).

Notably, Künzi et al. (2021) conducted a formal test of the pathway model using structural equation modelling and showing that childhood SEP predicted adulthood SEP, and adulthood SEP subsequently predicted later-life memory. Brewster et al. (2014) provided evidence for a pathway model in which adulthood literacy fully mediated the association between childhood SEP and later-life cognitive trajectories. Chen (2016) found support for a pathway between country recessions at the time of birth and later-life cognition.

#### 3.5.4. Accumulation model

Fifteen studies supported the accumulation model (Aartsen et al., 2019; Chen, 2016; Cheval et al., 2019; Ding & He, 2021; Greenfield et al., 2020; Landy et al., 2017; Leist et al., 2014; Luo & Waite, 2005; Lyu & Burr, 2016; Marden et al., 2017; Moorman et al., 2019; Peterson et al., 2021; Selvamani & Arokiasamy, 2021; Ye et al., 2022; Zeng et al., 2022). These studies showed that socioeconomic disadvantages affected later-life cognition at all points in life and that childhood adversities may accelerate or even worsen in middle age and older age. This resulted in a cumulative negative effect on later-life cognition levels and trajectories. Seven of these studies showed that cumulative socioeconomic positions or mobility in the socioeconomic positions over the life course affected later-life cognition (Leist et al., 2014; Luo & Waite, 2005; Lyu & Burr, 2016; Marden et al., 2017; Peterson et al., 2021; Selvamani & Arokiasamy, 2021; Zeng et al., 2022). We did not find any studies examining intergenerational transmission.

Importantly, the study by Luo and Waite (2005) demonstrated how sensitive period, pathway, and accumulation models can overlap. In their study, childhood SEP affects cognition in later life, and the impact of childhood SEP on education and income in adulthood explained a large share of this effect. Meanwhile, these three measures of SEP were directly related to levels of cognition, which supports the accumulation model.

#### 3.5.5. Place and time

In addition to SEP related to different life course periods, some studies also observed differences in SEP associations with cognition in later life by place and time. These results emphasize that the social, economic, and cultural contexts of different times and places may distinctly influence people’s experiences, opportunities, and life trajectories. Reynolds et al. (2022), for example, found better levels of cognitive function and a slower decline for respondents who grew up in the South of the United States, compared to the rest of the country. Wilson et al. (2005) reported a significant positive association between SEP at the county level and levels of later-life cognition but not with trajectories. Zhang et al. (2008) reported similar results. They found that being born in an urban was associated with lower odds of cognitive impairment, compared to being born in a rural area. Moorman et al. (2019) showed a modest but positive effect of attending an advantaged secondary school on levels of later-life cognition. Leist et al. (2021) found similar results in Europe that higher inequality of educational opportunity relates to lower levels of cognition. Faul et al. (2021) found a differential effect of education on cognitive decline between the United Kingdom (significant association) and the United States (no significant association).Peterson et al. (2021), however, showed no moderation of childhood residence on the effect of childhood and older-age SEP. Also, two studies did not find evidence of any country or regional differences (Cermakova et al., 2018; Wahrendorf et al., 2020). Some studies supported SEP associations by place and time (Chen, 2016; Fritze et al., 2014; Leist et al., 2014; Maharani, 2020; Muhammad et al., 2022; Reynolds et al., 2022).

## 4. Discussion and implications

### 4.1. Cognitive levels in the second half of life

The cross-sectional association between any measure of SEP and cognitive function in older age (Yaple & Yu, 2019), as well as the association between educational achievement and cognitive function in later life (Valenzuela & Sachdev, 2006), were known and documented by systematic reviews and meta-analyses, and were confirmed in our review based on longitudinal and general population studies. Beyond these two time points in the life course (young adulthood and older ages), our review also found associations between SEP at several life course periods – childhood or adolescence, young adulthood, middle age, and older age – and levels of cognitive function in later life. In other words, our review showed that SEP throughout the major life periods was associated with levels of cognitive function in later life.

In terms of SEP indicators, most of the included studies measured SEP using individual dimensions (e.g., income or occupation). According to the four dimensions of SEP – education, income, occupation and wealth (Hällsten & Thaning, 2022), we found that the associations with global cognition were generally consistent across life course periods. Higher education, whether parental or individual, consistently emerged as a strong predictor of better levels of global cognition in later life. Similarly, higher income across childhood, middle age, or older age was positively associated with cognitive levels. Non-manual occupations, whether parental or individual, were broadly linked to better levels of global cognition, particularly in childhood, middle age, and older age. For wealth, studies focusing on older age consistently demonstrated that higher wealth was associated with better cognitive levels. While findings varied slightly by life course periods, the overall pattern underscores a reliable protective relationship between advantaged SEP and better global cognitive levels, evident across all types of SEP resources.

Across specific cognitive domains, associations between life course SEP and levels of global cognition were similarly consistent across SEP dimensions, with advantaged SEP being protective for memory, executive functioning, processing speed, sensation/perception, motor skills, and language skills. However, the number of studies examining each SEP dimension by life course period and levels of cognitive domains was limited for certain domains, such as sensation/perception and motor skills.

Importantly, and first, these associations were robust to confounding factors, potential mediators in the causal pathway (between exposure and outcome) and other competing risk factors. We observed indeed that several studies adjusted for factors that could potentially account for the link between life course SEP and cognitive status. For example, these associations were generally robust to adjustment with childhood health, a factor that has been shown to influence childhood and adolescent SEP (Hale et al., 2015) as well as cognition in mid-life and older age (González et al., 2013). Second, these results were robust to the potentially mediating factor of chronic conditions in later life, known to be associated with cognitive function (Cheng et al., 2012; Hill et al., 2021). Third, two studies (Ritchie et al., 2016; Zaninotto et al., 2018) adjusted for dementia and most studies excluded participants with cognitive impairment or dementia.

This review thus documents a substantial and growing body of evidence that past SEP influences cognitive levels in the second half of life and that this influence of socioeconomic background goes back to childhood. SEP operates through mechanisms that afford disparate access to material, social, and cultural resources and opportunities (Bartley, 2016; Bourdieu, 1986; Weber, 1946). Two primary mechanisms are likely at play: first, the cognitive stimulation linked with higher SEP at various stages of the life course, such as growing up in intellectually stimulating environments, pursuing extended education, having demanding high-skill jobs, and participating in cognitively enriching leisure activities. Second, individuals with lower SEP are disproportionately exposed to environmental and psychosocial stressors, which can negatively impact cognitive function.

This review also found that the association of life course SEP, and in particular childhood SEP, was similar across cognitive domains, and not specific to any one cognitive domain. This supported the evidence that individual differences in different cognitive domains are correlated (Tucker-Drob, 2009). However, many of the included studies used a measure of global cognition, which limited the ability to draw firm conclusions. If this result were to be confirmed by future evidence syntheses, it would mean that the two mechanisms provided by Ursache and Noble (2016) are at play (i.e., stimulating language cortex and stressor exposure).

### 4.2. Cognitive trajectories in the second half of life

Some individuals experience a deterioration in cognitive function earlier and more rapidly than what is expected for their SEP (Murman, 2015). The present study examined whether this might be the case depending on the life course SEP. With regard to cognitive decline, the evidence of the association between life course SEP and rates of decline was mixed, as well as across SEP dimensions. When examining the association between childhood SEP and cognitive decline, 12 studies observed a significant direct link. However, the association was sustained only in five studies after adjusting for SEP in young adulthood or middle age. Therefore, the evidence is insufficient to conclude that childhood SEP influences cognitive decline in older ages. This conclusion is also reinforced by the fact that, across domains of cognitive decline, our review found inconclusive findings. Three studies have found that cognitive decline by early-life SEP strata is more pronounced among women than men. At most, more studies examining this association separately for women and men could be valuable.

Although SEP in middle and older age has a less definitive influence on cognitive aging, some evidence suggests its role in shaping cognitive trajectories. Faul et al. (2021) and Marden et al. (2017) found associations between social mobility, late-life income, and memory decline, with Marden highlighting that late-life income was a stronger determinant than education. Ye et al. (2022) observed an association between old-age household consumption and global cognition scores, though inconsistently. Aartsen et al. (2019) reported that higher financial resources in old age were linked to steeper cognitive decline, potentially explained by Stern’s cognitive reserve hypothesis (Stern, 2002), where greater cognitive stimulation offers initial protection and accrue over the life course but leads to sharper declines later. These findings, while intriguing, are based on limited evidence, and the weight of studies suggests that life course SEP more strongly affects cognitive levels than rates of decline. Further research is needed to refine these conclusions.

Our review concludes that life course SEP influences levels of cognitive function in older age, but that it likely does not influence cognitive evolution (although this remains to be confirmed). One implication of this conclusion is that the influence of SEP is mostly structural – it generates cross-sectional differences, probably in early- or middle-life, in the level of functioning but has little influence on cognitive aging (slopes of cognitive functions mostly parallel by SEP categories). Whereas these differences in cognitive function were present at the baseline of a longitudinal study, the structural difference probably originated before the participants were included in the surveys (i.e., before the second half of life). There are several possible explanations for this finding.

First, SEP, understood as a social status structure by differential access to resources (material, social and cultural), is likely to shape the developmental periods of life (childhood, adolescence, young adulthood) as well as during the period of building family life and professional career. However, in later life the role of resources as an influence on cognitive aging may become less important. In other words, the main influence of resources on cognitive development and reserve likely precedes older age.

Second, given that cognitive function in non-institutionalized populations tends to evolve slowly between adulthood and older age, some of the included studies are limited in several ways such as insufficient statistical power to detect intra-individual differences in change, too few longitudinal waves, a relatively short follow-up, and statistical methods used that do not allow for the most appropriate assessment of change (Ghisletta et al., 2019).

### 4.3. Life course models

For each study, our scoping review also assessed whether any of the four life course models were (a) supported explicitly or (b) provided some evidence consistent with an interpretation of influence—when the empirical evidence supporting the life course models was interpreted by the authors of this review without it being an explicit aim of the examined studies.

Most of the studies included in this review provided some evidence consistent with the sensitive period model. The sensitive period model assumes that SEP can independently influence later-life cognition in all periods of the life course prior to older age, and in particular in developmental periods such as childhood, adolescence and young adulthood. In support of the sensitive period model, we know that childhood cognitive development influences the peak level of cognitive function in young adulthood and the “baseline” level of aging-related cognitive decline (Tucker-Drob, 2019). There is a body of evidence showing that children born in disadvantaged households have a lower chance of achieving the best perinatal, developmental, and social outcomes. Indeed, childhood SEP is cross-sectionally associated with volume and cortical surface area of frontal regions, and amygdala, hippocampal, and striatal volume, and longitudinally associated with the development of grey matter structure in youth (Rakesh & Whittle, 2021). Similar results have been observed later in adolescence, a life course period during which different neurological functions have been linked with SES, and this is with regard to different conceptualizations and coding of SES (Buckley et al., 2019).

Despite this support, significant gaps remain in our understanding of the underlying mechanisms that make childhood a sensitive period for cognitive function later in life. One major question that remains unresolved is whether the influence of childhood SEP on later-life cognitive outcomes is primarily driven by exposure to a stimulating or unstimulating social environment, which could affect cognitive development directly, or whether it is more strongly linked to increased exposure to various forms of stress – such as psychosocial stress, adversity, and environmental stress in the neighbourhood – that are more common in disadvantaged settings. It is also possible that both mechanisms are at play, interacting in complex ways that have yet to be clarified. Furthermore, the relative contribution of these mechanisms across different stages of development is not well understood. Does the impact of early-life SEP stem more from the accumulation of cognitive stimulation, or from repeated or chronic stress exposure, or from a combination of these factors? These questions highlight the limitations of our results and underscore the need for future research to disentangle these mechanisms, which is crucial for understanding how early-life conditions shape cognitive trajectories into old age.

This review observed substantial support for the pathway model: 23 studies provided some evidence that education is a pathway (and potential mediator) in the influence of childhood or adolescent SEP on later-life cognition. The pathway model suggests that growing up in a family with poor socioeconomic conditions may jeopardize the child’s educational achievement. This is the pathway most often examined in the literature, meaning that we would observe a “cognitive gradient” in older ages between levels of education. As an illustration, low childhood SEP also leads to higher body weight which can impact educational achievement and well-being (Langford et al., 2022; Sirin, 2005). As a result, in early adulthood, the volume of the hippocampus is significantly lower in individuals with lower education compared to individuals with higher education (Noble et al., 2012).

While the pathway model provides a valuable framework for understanding how SEP influences cognition through education, it remains unclear how the two mechanisms – intellectual environment and stress – operate within this sequence of life course events. Specifically, the model assumes a linear progression where early-life SEP impacts education, which in turn affects cognitive outcomes, but it does not fully account for how variations in intellectual stimulation or stress exposure at each stage of life might modify this pathway. Moreover, how do these mechanisms interact with other SEP-related factors, such as access to resources or social support, throughout the life course? The current literature provides limited insight into these complex interactions, suggesting a need for more comprehensive research that integrates these mechanisms into the pathway model.

Fifteen studies supported the accumulation model which assumes that the influence of SEP across life course periods can be additive, meaning that SEP in two or more life course periods (prior to older age) can influence cognition in later life. It is plausible that evidence on the relationship between SEP and cognition can be supported by both the pathway and accumulation models, but the latter has stronger data requirements to test accumulation relationships over the life course or across domains of SEP exposures (Ferraro & Morton, 2018).

However, while the accumulation model provides a framework for understanding how SEP influences cognitive function, there are limitations in the current evidence, particularly regarding the role of the two mechanisms – exposure to intellectual environments and exposure to stress – that link SEP to cognition. Most studies do not sufficiently explore how these mechanisms operate in tandem or interact over multiple life stages. Additionally, the accumulation model’s emphasis on additive effects may oversimplify the complex, dynamic nature of these mechanisms, which may not merely accumulate but interact in non-linear ways. This suggests a need for more nuanced research that integrates these mechanisms into the accumulation framework to better capture the multifaceted impact of SEP on cognitive aging.

Last, this review found no support for the critical period model. In other words, the association between poor socioeconomic conditions in one developmental period and cognitive health in later life was modified by later socioeconomic conditions in the life course, such as in young adulthood, middle life, or older age. This suggests that no single life period entirely determines cognitive function in older ages. Nevertheless, we advise against the outright dismissal of this model. The model may be applicable in specific contexts where extreme poverty during early childhood – particularly during the first 1000 days of life – results in severe malnutrition and stunted cognitive development (Pizzol et al., 2021; Kirolos et al., 2022). Individuals affected in this way may have higher child and premature mortality (Pelletier, 1994) and are often not included in longitudinal studies on individuals aged 50 and over (a phenomenon known as health selection), the type of studies typically included in this review. Furthermore, our review excluded studies on patient populations, including those with psychiatric disorders or a diagnosis of dementia.

**Fig. 2.**
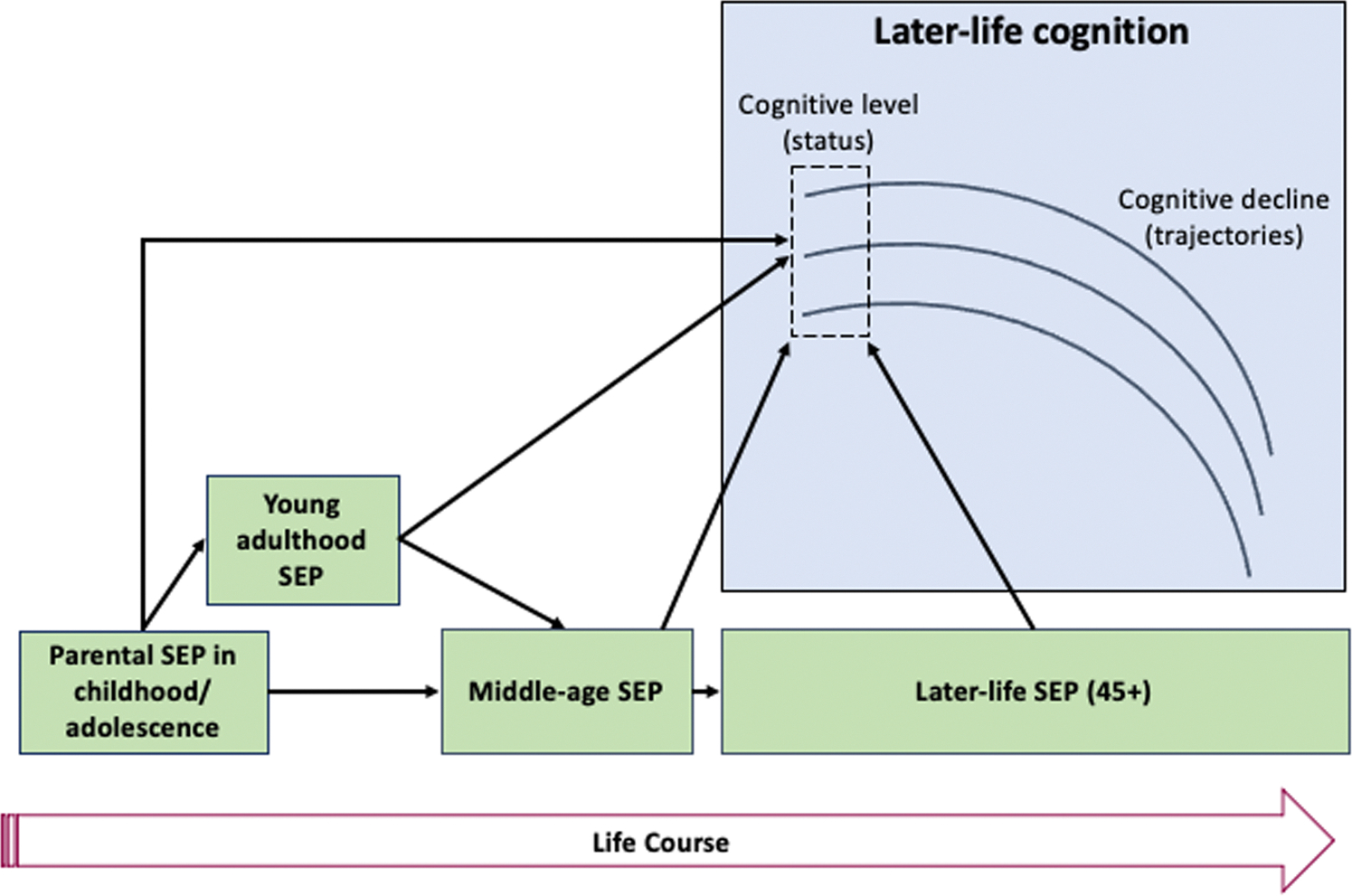
A heuristic framework based on the findings from the scoping review to illustrate mechanisms underlying the relationships between life course SEP and later-life cognition. Socioeconomic Position (SEP) across the life course is represented in green boxes, and later-life cognition in the blue box. Solid arrows indicate SEP effects supported by robust evidence identified in this review, predominantly for cognitive level (status). The lack of consistent evidence regarding cognitive decline (trajectories) has led to the omission of corresponding arrows.

Our review thus found substantial support for the sensitive period model and relatively substantial support for the pathway and accumulation models, observations that suggest that SEP during child developmental and later stages of the life course is a driver of difference in cognitive function in older ages. To summarize the life course influences based on our findings, we present Fig. 2 and begin by recognizing the structural influence of parental SEP during childhood and adolescence. Further research is needed to draw definitive conclusions on the applicability of these life course models. Our scoping review provides no evidence to support the critical period model, but evidence in support of the remaining three life course models. We also conclude that these three models are not mutually exclusive but complementary and intertwined (Wagner et al., 2022). To advance our understanding of life course influences on cognitive aging, future research should test these life course models simultaneously within the same studies. Integrating the sensitive period, pathway, and accumulation models in a single study could offer a comprehensive view of how these frameworks interact. This approach would allow for a more nuanced explanation of how life course SEP influences cognitive health in old age.

Furthermore, it should be noted that the life course models tested in this scoping review are not exhaustive, as we did not review the social mobility model. In addition, one theoretical framework warranting consideration is the reserve or resilience model, which was initially outlined by Ben-Shlomo et al. (2016) and subsequently expanded by Cullati et al. (2018). Independently but with a similar conceptual approach, Sabayan et al. (2023) proposed a brain resilience model within a life course framework. The reserve model posits that an individual’s health trajectory results from successive adaptations to micro-shocks or daily exposures (e.g., infections, environmental exposures, or psychosocial stressors) throughout the life course. Over time, the cumulative wear-and-tear or resources depletion may diminish the ability to adapt cognitively, leading to increased vulnerability and less effective recovery. Integrating such framework could provide additional insights into how life course SEP shapes cognitive health in older age, particularly through dynamic processes of adaptation and resilience.

Moreover, there is a pressing need for research that spans diverse geographical and cultural contexts, including low- and middle-income countries. Such studies could provide insights into how SEP influences cognitive outcomes in varying socioeconomic environments and help identify region-specific factors that may affect cognitive aging.

While these life course models help elucidate how SEP influences cognitive health, they also underscore the importance of integrating specific mechanisms by which SEP may impact cognition, such as intellectual environment and stress exposure. The interplay between these mechanisms and the life course models will offer a more nuanced understanding of SEP’s influence on cognitive function. Although the mechanisms of intellectual environment and stress are relevant across different life course periods, their effects may vary depending on the life course model being applied. This highlights the need for future research to first investigate the interaction between these SEP mechanisms and their impact on cognitive function across various life stages, and second, to explore how these mechanisms operate within different life course models more comprehensively (Blane et al., 2013).

The findings from this review suggest that interventions aimed at improving cognitive health in later life could consider the life course perspective, in particular the sensitive period, pathway and accumulation models. Policies that target SEP early in life, such as improving access to education and stimulating environments in childhood, could have long-term benefits for cognitive health. In addition, policies that address socioeconomic inequalities across the life course, including adolescence and adulthood, may help mitigate the cumulative negative effects of low SEP on cognitive aging. Moreover, given the interaction between intellectual environment and stress exposure, health promotion efforts could also focus on reducing stress and promoting cognitive stimulation at all stages of life, which may be particularly effective in enhancing cognitive resilience in older ages. Our findings highlight the need for comprehensive life-course-oriented policy strategies that address SEP disparities to improve cognitive outcomes across populations. Further research is needed to test the effectiveness of these strategies.

### 4.4. Limitations of the scoping review

This scoping review has several limitations. First, as the aim was to ensure comprehensive coverage, the articles retrieved are heterogeneous in terms of SEP exposures and cognitive outcomes. For the exposure, we searched for a diversity of socioeconomic position measures and studies with new SEP exposures were included to reflect the diversity of approaches. Furthermore, the timing (within and between life course periods) of the measurement of socioeconomic position was not similar in all studies. Moreover, different SEP measures show different levels of stability. For example, income is dynamic over the life course and the effects of income on health can also fluctuate over time (Foverskov et al., 2020). This represents a challenge to test different life course models and make the distinction between these models. Also, we cannot exclude the possibility that key SEP exposures were not included in this review. While variability in SEP operationalizations does not always imply inconsistencies – e.g., the level of education consistently shows significant associations with cognitive outcomes (Maccora et al., 2020) – there is a need for further research. Specifically, the impact of subjective SEP, which has been shown to be a good predictor of subjective well-being (Tan et al., 2020), warrants more exploration. Subjective SEP reflects individuals’ perceptions of their social standing and may offer unique insights into the relationship between SEP and cognition that are not captured by objective measures alone.

For the outcome, all studies included objective measurements of cognitive function, but cognitive tests varied by studies (Mini-Mental State Examination (Folstein et al., 1975), Telephone Interview of Cognitive Status, etc.). All of these tests were implemented in the context of general population surveys and for screening purposes, and therefore cannot be considered as diagnostic tests, like the Mini-Mental State Examination (Mitchell, 2009). For these reasons and given that the objective of this scoping review was to gather exposure variability, the positivity assumption regarding exposure and outcome cannot be guaranteed. This limitation reduces the ability to draw causal conclusions from our results.

Second, although our literature search was broad, it was non-systematic, which may have led to the omission of some relevant studies and affected the representativeness of our findings. Therefore, we recommend that our findings are viewed alongside relevant reviews reporting on inequalities in cognitive aging in older age (Chen et al., 2022; Kramer et al., 2004; Meng et al., 2022; Xu et al., 2016; Zhao et al., 2021).

Third, all but four of the included study datasets (LBC, NSHD, SATSA, and WLS) had retrospective data on socioeconomic indicators, generally for the childhood and young adulthood periods. The reliability of retrospective responses concerning the highest level of education and other past SEP indicators achieved is generally satisfactory (Brown, 2014; Lacey et al., 2012). However, for childhood, retrospective data might have led to a lower response rate (von Fintel & Posel, 2016) and recall bias regarding living conditions, like childhood social class (Batty et al., 2005). Nevertheless, methodological evidence on retrospective measures of childhood socioeconomic conditions suggested an acceptable level of validity (Brown, 2014; Havari & Mazzonna, 2015; Jivraj et al., 2020; Lacey et al., 2012; McKenzie & Carter, 2009) and it has been noted that validity of retrospective data is sensitive to the types of socioeconomic information collected, with domestic water facilities or father’s occupation reaching satisfactory reliability (Berney & Blane, 1997).

Fourth, our findings were largely based on studies from Europe and the USA, which prevents the generalisability of these results to middle and low-income countries. This limitation is largely due to the scarcity of longitudinal data infrastructures in these regions, resulting in fewer studies. Furthermore, it is plausible that the generalisability of our findings is not universal due to unmeasured cultural and socioeconomic differences. Nevertheless, we cannot exclude the possibility of transposing our results in the latter two groups of countries when SEP is defined broadly as a global, non-specific measure (low vs. high SEP). Additionally, it would be interesting to explore national and cultural differences in the SEP and cognition association in future research.

Fifth, other factors can affect cognitive decline in older age, such as widowhood (Singham et al., 2021) and healthcare access (Mullins et al., 2021). Of note, widowhood could be on the causal pathway between socioeconomic living conditions in childhood and cognition in older age, and mediate the association observed in this review.

Sixth, the social mobility model, which focuses on how individuals move between different SEP groups across their lives, was integrated within the broader framework of the accumulation life course model rather than being treated as a separate model. Although including the social mobility model might have expanded the scope of the review by addressing how changes in social class affect life course experiences, we have reported associations related to social mobility whenever they were considered in the studies included in this review.

Seventh, the results from this scoping review may be impacted by a potential selection bias due to unsatisfactory participation rate at baseline and important follow-up attrition in the included studies. Selection bias occurs when the participants included in the study are not representative of the general population. Generally, participants in better health condition tend to stay in the study, while those in poorer health tend to leave the study. As a result, the findings may not accurately reflect the true association between exposure and outcome in the broader population.

Eighth, publication bias may affect our results. Publication bias occurs when studies showing significant associations are more likely to get published, and we cannot exclude the possibility that research groups may have refrained from publishing papers with a non-significant association between life course socioeconomic position and cognitive outcomes in old age. As a result, the available literature may be biased, potentially exaggerating the perceived strength or direction of an association. This potential bias is a reminder that the results of this scoping review should be interpreted with caution.

Finally, we have not assessed the methodological quality of the included studies meaning that the review potentially included low-quality studies with risk of bias, which could increase the risk of synthetizing biased findings. However, the approach we used to extract information from the included studies enabled us to consider aspects of study quality. Specifically, our data extraction considered three factors that could confound or mediate the association between SEP and cognition: childhood health indicators, old-age chronic conditions, and genetic factors. Notably, 12 studies adjusted for childhood health conditions and 17 for later-life chronic conditions, which suggest some robustness in our findings. Nonetheless, the possibility of residual confounding remains, which could lead to misleading results. This scoping review should be viewed as an initial exploration into the complex relationship between life-course SEP and cognition. Future research could benefit from more focused systematic reviews that target specific SEP dimensions across the life course and specific cognitive domains in old age.

## 5. Conclusion

The results of this review highlight the importance of socioeconomic factors across the life course in relation to cognitive function in later life. It also provides support for the sensitive period, pathway, and accumulation models, but not for the critical period model. Future research on cognitive function in older adults should also consider the potential interactions (timing and sequence) between exposures over several periods of life, the stability and change of SEP, and interventions should consider SEP across the life course of the target individuals, as well as across different contexts. One potential implication of our conclusions is that stronger policies on early childhood education and access to economic resources may mitigate the impact of socioeconomic factors on cognitive outcomes in life course. In a field of research where complexity is omnipresent (Rod et al., 2023), this review calls for more focused systematic reviews that target specific SEP dimensions across the life course and specific cognitive domains in old age.

## Supplementary Material

Supplementary

## Figures and Tables

**Table 1:** Overview of studies included in the scoping review (n = 42).

First author, year	Reference location	Data^a^ (baseline-end year), study design, sample size^b^	Mean age^c^, age range^d^	Cognitive domains^e^
Everson-Rose, 2003	U.S. (Chicago)	CHAP (1993–2003), older age cohort, n = 4398	M= 74, 65 +	Global cognition
Luo, 2005	U.S.	HRS (1992–1998), older age cohort, n = 19,949	M= 67, 50 +	Memory; executive functioning
Wilson, 2005	U.S. (Chicago)	CHAP (1993–1997), older age cohort, n = 4392	M= 74, 65 +	Global cognition
Zhang, 2008	China	CLHLS (1998–2000), older age cohort, n = 6475 or 8444 (across models)	M= 90 (women), M= 93 (men), 80–105	Global cognition
Brown, (2010)	U.S.	HRS (1995–2006), older age cohort, n = 30,896	M= 75, 59–83	Global cognition
Jefferson, 2011	U.S. (Chicago)	RMAP (2002–2008), older age cohort, n = 951	M= 79, 54–100	Global cognition; memory; processing speed; sensation/perception
Barnes, 2012	U.S. (Chicago)	CHAP (1993–2000), older age cohort, n = 6158	M= 75, 65 +	Global cognition
González, 2013	U.S.	HRS (1998–2010), older age cohort, n = 8833	M= 74, 65 +	Global cognition
Hurst, 2013	UK	NSHD (2006–2011), birth cohort, n = 2229	M=NA, 0–64	Memory; processing speed; motor skills
Brewster, 2014	U.S. (Northern California)	UCD ADC (2001–2010), older age cohort, n = 333	M= 74, 60 +	Global cognition; memory; executive functioning
Fritze, 2014	Europe	SHARE (2004 or 2006/2007), older age cohort, n = 7935	M=NA, 60–80 +	Global cognition
Leist, 2014	Europe	SHARE (2004 or 2006/2007), older age cohort, n = 12,020	M= 63, 50–74	Global cognition
Chen, (2016)	China (Taiwan)	TLSA (1989), older age cohort, n = 4328	M= 64, NA	Global cognition
Lyu, 2016	U.S.	HRS (1998–2010), older age cohort, n = 9407	M= 75, 65 +	Global cognition
Marden, 2016	U.S.	HRS (1998–2012), older age cohort, n = 10,781	M= 66, 50 +	Memory
Ritchie, 2016	Scotland	LBC (1936–2014), birth cohort, n = 552	M= 70, 68–78	Sensation/perception; language/verbal skills; memory; processing speed
Staff, 2016	Scotland	ABC 1936 (1936–2014), birth cohort, n = 478	M=NA, 63–78	Global cognition
Ericsson, 2017	Sweden	SATSA (1984–2012), twin cohort, n = 859	M= 70, 50–96	Global cognition; memory; processing speed; language/verbal skills
Landy, 2017	UK	NSHD (1946–2011), birth cohort, n = 2293; WHITEHALL (1985–2009), occupational cohort, n = 3550	M=NA, 0–53 (NSHD), 0–74 (WHITEHALL)	Language/verbal skills
Cermakova, 2018	Europe	SHARE (2004–2015), older age cohort, n = 20,244	M= 71, 50 +	Global cognition; memory; language/verbal skills
Zaninotto, 2018	UK	ELSA (2002–2011), older age cohort, n = 11,391	M= 65, 50 +	Global cognition; memory; processing speed; executive functioning
Aartsen, 2019	Europe	SHARE (2004–2015), older age cohort, n = 24,066	M= 62, 50–96	Memory; language/verbal skills
Cheval, 2019	Europe	SHARE (2004–2015), older age cohort, n = 23,344	M= 64, 50–96	Memory; language/verbal skills
Moorman, 2019	U.S.	WLS (2004–2011), school cohort, n = 3012	M= 72, 65–72	Memory; language/verbal skills
Greenfield, 2020	U.S.	WLS (1957–2011), school cohort, n = 3706	M= 72, NA	Language/verbal skills; executive functioning; memory
Maharani, (2020)	Indonesia	IFLS (2014/2015), older age cohort, n = 6676	M= 60, 50 +	Global cognition
Wahrendorf, 2020	Europe & UK	SHARE & ELSA (2002–2015), older age cohorts, n = 22,620	M= 65, 50–85	Memory
Yang, 2020	China	CHARLS (2011–2015), older age cohort, n = 16,258	M= 59, 45–101	Global cognition; memory
Ding, 2021	China	CHARLS (2011–2015), older age cohort, n = 23,807	M= 59, 45 +	Memory; executive functioning
Faul, 2021	U.S. & UK	HRS (1998–2014) and ELSA (2002–2012), older age cohorts, n = 23,229 (HRS), n = 6008 (ELSA)	M= 63 (HRS), M= 61 (ELSA), 50 +	Memory
Kunzi, 2021	Switzerland	VLV (2011–2017), older age cohort, n = 993	M= 81, 70–103	Memory
Lee, 2021	U.S.	NSHAP (2015/2016), older age cohort, n = 3361	M= 64, 57–85	Global cognition
Leist, 2021	Europe	SHARE (2004–2017), older age cohort, n = 46,972	M= 51–67 (across countries), 50–76	Memory; language/verbal skills
Peterson, 2021	U.S.	KHANDLE (2017/2018), older age cohort, n = 1353	M= 75, 65 +	Memory; executive functioning
Selvamani, 2021	India & China	SAGE (2007/2010), older age cohort, n = 6560 (India), n = 13,106 (China)	M= 61.5 (India) M= 62.6 (China), 50 +	Global cognition
Wolfova, 2021	Europe	SHARE (2004–2015), older age cohort, n = 84,059	M= 64, 50 +	Memory; language/verbal skills
Ford, 2022	UK	ELSA (2006/2007), older age cohort, n = 4553	M= 62–65, 50–80	Memory
Muhammad, 2022	India	LASI (2017/2018), older age cohort, n = 31,464	M=NA, 60 +	Global cognition
Reynolds, 2022	U.S.	HRS (2010–2016), older age cohort, n = 8299	M= 76, 65 +	Global cognition
Ye, 2022	China	CHARLS (2011–2015), older age cohort, n = 11,644	M= 59, 45 +	Global cognition
Zeng, 2022	U.S.	HRS (1998–2016), older age cohort, n = 8376	M= 74, 50 +	Memory; executive functioning
Zhou, 2022	China	CHARLS (2018), older age cohort, n = 16,286	M= 62, 45–118	Memory; executive functioning

Note.

NA = not available.

a ABC 1936=Aberdeen Birth Cohort Study 1936, CHAP = Chicago Health and Aging Project, CHARLS = China Health and Retirement Longitudinal Study, CLHLS = Chinese Longitudinal Healthy Longevity Survey, ELSA = English Longituinal Study of Ageing, HRS = Health and Retirement Study, IFLS= Indonesia Family Life Survey, KHANDLE = Kaiser Healthy Aging and Diverse Life Experiences Cohort, LASI=Longitudinal Ageing Study in India, LBC = Lothian Birth Cohort, NSHAP = National Social Life, Health, and Aging Project, NSHD = National Survey of Health and Development, RMAP = Rush Memory and Aging Project (Chicago), SAGE=WHO Study on Global AGEing and Adult Health, SATSA = Swedish Adoption Twin Study of Aging, SHARE = Survey of Health, Ageing and Retirement in Europe, TLSA = Taiwan Longitudinal Study on Aging, UCD ADC = UC Davis Aging Diversity Cohort, VLV = Vivre-Leben-Vivere, WHITEHALL = Whitehall II Occupational Cohort Study, WLS = Wisconsin Longitudinal Study.

b Sample size in the final model.

c Mean age in the final model.

d Age range of the dataset/sample.

e Classification of cognitive domains by Harvey (2019).

**Table 2:** Summary of associations between socioeconomic position (SEP) and cognitive levels and trajectories, by life course SEP periods and cognitive domains^a^ (n = 42).

First author, year	Associations between life course SEP periods and cognitive levels	Associations between life course SEP periods and cognitive trajectories
Everson-Rose, 2003	+ Childhood/adolescent SEP	× Childhood/adolescent SEP
Luo, 2005	Across cognitive domains: + Childhood/adolescent SEP + Education + Older-age SEP + *Cumulative SEP* + *SEP mobility*	/
Wilson, 2005	+ Childhood/adolescent SEP, mediated by adult SEP + *County SEP*	× Childhood/adolescent SEP × County SEP
Zhang, 2008	+ Childhood/adolescent SEP + Middle-age SEP (only for women in full model)	+ Childhood/adolescent SEP × Middle-age SEP
Brown, (2010) ^b^	+ Childhood/adolescent SEP	× Childhood/adolescent SEP
Jefferson, 2011	Across cognitive domains: + Childhood/adolescent SEP + Education + Middle-age SEP (except memory and sensation/perception) + Older-age SEP (except memory and sensation/perception)	/
Barnes, 2012	+ Childhood/adolescent SEP, mediated by education	- Childhood/adolescent SEP, not mediated by education (only for African Americans)
González, 2013	+ Childhood/adolescent SEP + Education + Older-age SEP	× Childhood/adolescent SEP × Education × Older-age SEP
Hurst, 2013	Across cognitive domains: + Childhood/adolescent SEP + Adult SEP + Education	/
Brewster, 2014	+ Childhood SEP vs. both domains (global cognition not tested) × Education vs. memory + Education vs. executive functioning (only for nonLatino whites)	+ Childhood/adolescent SEP (only global cognition tested) × Education (only global cognition tested)
Fritze, 2014	+ Childhood/adolescent SEP + Education + *Macro-level SEP (economic boom)*	/
Leist, 2014	+ Childhood/adolescent SEP + Education - *Young adulthood SEP (white collar as first job)* - *Macro-level SEP (recessions)* - *Disadvantaged life-course occupational class mobility and working conditions*	/
Chen, (2016)	+ Childhood/adolescent SEP + Education - *Macro-level SEP (economic recession)*	/
Lyu, 2016	+ Childhood/adolescent SEP (except family financial status; mediated by adult SEP) + Education + Middle-age SEP + *Cumulative SEP* + *SEP mobility*	× Childhood/adolescent SEP (except mother’s education which is a protective factor) + Education × Middle-age SEP × *Cumulative SEP/SEP mobility*
Marden, 2016	+ Childhood/adolescent SEP + Education + Older-age SEP + *Life course trajectories of SEP*	+ Childhood/adolescent SEP + Education + Older-age SEP
Ritchie, 2016	Across cognitive domains: × Childhood/adolescent SEP (after including education and other covariates) + Education (except processing speed) × Middle-age SEP x Older-age SEP	Across cognitive domains: × Childhood/adolescent SEP × Education × Middle-age SEP × Older-age SEP
Staff, 2016	× Childhood/adolescent SEP (after including childhood ability and education) + Education + Middle-age SEP	× Childhood/adolescent SEP × Education × Middle-age SEP
Ericsson, 2017	Across cognitive domains: + Childhood/adolescent SEP + Education	Across cognitive domains: × Childhood/adolescent SEP × Education
Landy, 2017	+ Childhood/adolescent SEP + Education + Middle-age SEP	/
Cermakova, 2018	+ Childhood/adolescent SEP vs. memory & language/verbal skills	Across cognitive domains: × Childhood/adolescent SEP
Zaninotto, 2018	Across cognitive domains: + Childhood/adolescent SEP/education/wealth (except childhood SEP on processing speed among men)	Women: - Childhood/adolescent SEP vs. global cognition - Education vs. memory × Wealth vs. all domains Men: + Childhood/adolescent SEP vs. processing speed + Education vs. global cognition × Wealth vs. all domains
Aartsen, 2019	Across cognitive domains: + Childhood/adolescent SEP + Education + Middle-age SEP + Older-age SEP	Across cognitive domains: × Childhood/adolescent SEP (only for memory) - Childhood/adolescent SEP (only for language/verbal skills) + Education + Middle-age + Older-age
Cheval, 2019	Across cognitive domains: + Childhood/adolescent SEP + Education + Middle-age SEP + Older-age SEP	Across cognitive domains: × Childhood/adolescent SEP + Education (only for women) + Middle-age (only for memory among men)
Moorman, 2019	Across cognitive domains: + Childhood/adolescent SEP + Education + Middle-age SEP + *Secondary school advantages* (only for language/verbal skills)	Across cognitive domains: × Childhood/adolescent SEP × Education + Middle-age SEP (only for memory) - *Secondary school advantages* (only for memory)
Greenfield, 2020	Across cognitive domains: + Childhood/adolescent SEP + Education + Middle-age SEP	/
Maharani, (2020)	+ Childhood/adolescent SEP + Education + Older-age SEP	/
Wahrendorf, 2020	+ Middle-age SEP / Childhood/adolescent SEP, education, older-age SEP	/
Yang, 2020	Across cognitive domains: + Childhood/adolescent SEP + Education	Only for middle-aged adults: + Childhood/adolescent SEP across cognitive domains / Education
Ding, 2021	Across cognitive domains: + Childhood/adolescent SEP + Education + Older-age SEP	/
Faul, 2021	+ Childhood/adolescent SEP (only in ELSA) + Education + Older-age SEP	× Childhood/adolescent SEP + Education (only in HRS) + Older-age SEP (in ELSA) - Older-age SEP (in HRS)
Kunzi, 2021	× Childhood/adolescent SEP + Adulthood SEP × Older-age SEP	/
Lee, 2021	+ Childhood/adolescent SEP + Education + Middle-age SEP	/
Leist, 2021	Across cognitive domains: + Childhood/adolescent SEP + Education + Older-age SEP - *Inequality of educational opportunity* (except null effect on delayed recall among men)	Women and men: + *Inequality of educational opportunity* vs. memory (immediate recall); Only for women: - *Inequality of educational opportunity* vs. memory (delayed recall) & language/verbal skills
Peterson, 2021	Across cognitive domains: + Childhood/adolescent SEP (only if the SEP advantage was maintained into adulthood) + Education + Older-age SEP (only for memory) + *Life course SEP trajectories*	/
Selvamani, 2021	+ Childhood/adolescent SEP + Education + Middle-age SEP + Older-age SEP + *Life course SEP trajectories*	/
Wolfova, 2021	+ Childhood/adolescent SEP vs. memory × Childhood/adolescent SEP vs. language/verbal skills + Education vs. both domains	+ Childhood/adolescent SEP vs. memory (delayed recall) × Childhood SEP vs. memory (immediate recall) × Childhood/adolescent SEP vs. language/verbal skills
Ford, 2022	+ Childhood/adolescent SEP + Education + Middle-age SEP	/
Muhammad, 2022	+ Childhood/adolescent SEP + Education + Older-age SEP	/
Reynolds, 2022	+ Childhood/adolescent SEP	/
Ye, 2022	+ Childhood/adolescent SEP + Education + Older-age SEP	+ Childhood/adolescent SEP + Education + Older-age SEP
Zeng, 2022	Across cognitive domains: + Childhood/adolescent SEP + Education + Young adulthood SEP + Middle-age SEP - *Cumulative SEP disadvantages* - *SEP mobility*	For memory: + Childhood/adolescent SEP - Education - Young adulthood SEP - Middle-age SEP + *Cumulative SEP disadvantages* + *SEP mobility* For executive functioning: - Childhood/adolescent SEP + Education + Young adulthood SEP + Middle-age SEP - *Cumulative SEP disadvantages* - *SEP mobility*
Zhou, 2022	Across cognitive domains: + Childhood/adolescent SEP + Education + Older-age SEP	/

Note.

SEP = socioeconomic position.

Symbols for effect direction: + protective, - risk, × null (no statistically significant results), / not tested. If no subgroup (e.g., age group, ethnic group, etc.) is indicated, the results apply to the whole study population.

Text in Italic shows results of SEP indicators that are unique and not used by other studies.

a Cognitive domains for each study are reported in the last column of Table 1.

b Education and older-age income were included in the analysis, but results were not reported.

**Table 3:** Summary of associations between individual-level SEP indicators and global cognitive levels and trajectories, by life course SEP periods (n = 17).

Life course periods	Dimensions of SEP^a^	Global cognition	
		Levels^b^	Trajectories^b^
**Childhood/adolescence**	Education	+ (13)	+ (3)
			× (5)
	Income	+ (5)	+ (1)
			- a)
			× (1)
	Occupation	+ (7)	+ (1)
		× (1)	- (1)
			× (4)
	Wealth		
**Young adulthood**	Education	+ (9)	× (5)
	Income		
	Occupation	+ (1)	
	Wealth		
**Middle age**	Education		
	Income	+ (1)	
	Occupation	+ (3)	× (2)
	Wealth		
**Older age**	Education		
	Income	+ (5)	× (2)
	Occupation	+ (2)	
	Wealth	+ (3)	× (2)

SEP= socioeconomic position. Each SEP indicator reflects the parents’ characteristics when the life course period is childhood/adolescence and the individuals for the other periods.

a Dimensions of individual-level SEP refer to the “big four” model (Hällsten & Thaning, 2022).

b Symbols for effect direction: + protective, - risk, × null (not statistically significant). Each cell counts the studies which showed the abovementioned effect direction.

## Data Availability

Access to data requires contacting the last author. This scoping review was not registered in a review register.
