# Supplementary material for: Life course socioeconomic position and cognitive aging in later life: A scoping review
Source: Adv Life Course Res. Author manuscript; Available in PMC 2025 Jul 1. (PMC12124963; doi:10.1016/j.alcr.2025.100670)
Supplement: Supplementary [file NIHMS2075849-supplement-Supplementary.docx]

**Table S1. List of non-included studies**

|  | **References** | **Reasons for exclusion** |
| --- | --- | --- |
| 1 | Salthouse, T. A. (2014). Correlates of cognitive change. *Journal of Experimental Psychology. General*, *143*(3), 1026–1048. https://doi.org/10.1037/a0034847 | Not explicitly test life course models |
| 2 | Burr, J. A., Han, S. H., & Peng, C. (2020). Childhood friendship experiences and cognitive functioning in later life: The dediating roles of adult social disconnectedness and adult loneliness. *The Gerontologist*, *60*(8), 1456–1465. https://doi.org/10.1093/geront/gnaa055 | No focus on SEP indicators |
| 3 | Roberts, A. L., Sumner, J. A., Koenen, K. C., Kubzansky, L. D., Grodstein, F., Rich-Edwards, J., & Weisskopf, M. G. (2022). Childhood abuse and cognitive function in a large cohort of middle-aged women. *Child Maltreatment*, *27*(1), 100–113. https://doi.org/10.1177/1077559520970647 | No focus on SEP indicators |
| 4 | Gow, A. J., Johnson, W., Mishra, G., Richards, M., Kuh, D., Deary, I. J., & HALCyon Study Team. (2012). Is age kinder to the initially more able?: Yes, and no. *Intelligence*, *40*(1), 49–59. https://doi.org/10.1016/j.intell.2011.10.007 | No focus on SEP indicators |
| 5 | Foverskov, E., Glymour, M. M., Mortensen, E. L., Holm, A., Lange, T., & Lund, R. (2018). Education and cognitive aging: Accounting for selection and confounding in linkage of data from the Danish Registry and Survey of Health, Ageing and Retirement in Europe. *American Journal of Epidemiology*, *187*(11), 2423–2430. https://doi.org/10.1093/aje/kwy162 | Not explicitly test life course models |
| 6 | Burzynska, A. Z., Ganster, D. C., Fanning, J., Salerno, E. A., Gothe, N. P., Voss, M. W., McAuley, E., & Kramer, A. F. (2020). Occupational physical stress is negatively associated with hippocampal volume and memory in older adults. *Frontiers in Human Neuroscience*, *14*, 266. https://doi.org/10.3389/fnhum.2020.00266 | No focus on SEP indicators |
| 7 | Bloomberg, M., Dugravot, A., Dumurgier, J., Kivimaki, M., Fayosse, A., & Steptoe, A. (2021). Sex differences and the role of education in cognitive ageing: Analysis of two UK-based prospective cohort studies. *Lancet Public Health*, *6*(2), e106–e115. https://doi.org/10.1016/s2468-2667(20)30258-9 | Not explicitly test life course models |
| 8 | Smith, J. A., Kho, M., Zhao, W., Yu, M., Mitchell, C., & Faul, J. D. (2021). Genetic effects and gene-by-education interactions on episodic memory performance and decline in an aging population. *Social Science & Medicine*, *271*, 112039. https://doi.org/10.1016/j.socscimed.2018.11.019 | No focus on SEP indicators |
| 9 | Singh-Manoux, A., Kivimaki, M., Glymour, M. M., Elbaz, A., Berr, C., Ebmeier, K. P., Ferrie, J. E., & Dugravot, A. (2012). Timing of onset of cognitive decline: Results from Whitehall II prospective cohort study. *BMJ*, *344*, d7622. https://doi.org/10.1136/bmj.d7622 | No focus on SEP indicators |
| 10 | Beller, J., & Wagner, A. (2020). Loneliness and health: The moderating effect of cross-cultural individualism/collectivism. *Journal of Aging and* *Health*, *32*(10), 1516–1527. https://doi.org/10.1177/0898264320943336 | No focus on SEP indicators |
| 11 | Munoz, E., Sliwinski, M. J., Scott, S. B., & Hofer, S. (2015). Global perceived stress predicts cognitive change among older adults. *Psychology and* *Aging*, *30*(3), 487–499. https://doi.org/10.1037/pag0000036 | No focus on SEP indicators |
| 12 | Steptoe, A., & Zaninotto, P. (2020). Lower socioeconomic status and the acceleration of aging: An outcome-wide analysis. *PNAS*, *117*, 14911–14917. https://doi.org/10.1073/pnas.1915741117 | - Not explicitly test life course models  - Not from a new country and dataset already included |
| 13 | Formánek, T., Csajbók, Z., Wolfová, K., Kučera, M., Tom, S., Aarsland, D., & Cermakova, P. (2020). Trajectories of depressive symptoms and associated patterns of cognitive decline. *Scientific Reports*, *10*(1), 20888. https://doi.org/10.1038/s41598-020-77866-6 | - No focus on SEP indicators  - Not from a new country and dataset already included |
| 14 | Chapman, B., Duberstein, P., Tindle, H. A., Sink, K. M., Robbins, J., Tancredi, D. J., Franks, P., & Gingko Evaluation of Memory Study Investigators. (2012). Personality predicts cognitive function over 7 years in older persons. *American Journal of Geriatric Psychiatry*, *20*(7), 612–621. https://doi.org/10.1097/JGP.0b013e31822cc9cb | No focus on SEP indicators |
| 15 | Amieva, H., Jacqmin-Gadda, H., Orgogozo, J. M., Le Carret, N., Helmer, C., Letenneur, L., Barberger-Gateau, P., Fabrigoule, C., & Dartigues, J. F. (2005). The 9 year cognitive decline before dementia of the Alzheimer type: A prospective population-based study. *Brain*, *128*(5), 1093–1101. https://doi.org/10.1093/brain/awh451 | No focus on SEP indicators |
| 16 | Tucker-Drob, E. M., Johnson, K. E., & Jones, R. N. (2009). The cognitive reserve hypothesis: A longitudinal examination of age-associated declines in reasoning and processing speed. *Developmental Psychology*, *45*(2), 431–446. https://doi.org/10.1037/a0014012 | Not explicitly test life course models |
| 17 | Lang, I. A., Llewellyn, D. J., Langa, K. M., Wallace, R. B., Huppert, F. A., & Melzer, D. (2008). Neighborhood deprivation, individual socioeconomic status, and cognitive function in older people: Analyses from the English Longitudinal Study of Ageing. *Journal of the American Geriatrics Society*, *56*(2), 191–198. https://doi.org/10.1111/j.1532-5415.2007.01557.x | - Not explicitly test life course models  - Not from a new country and dataset already included |
| 18 | Kuchibhatla, M., Hunter, J. C., Plassman, B. L., Lutz, M. W., Casanova, R., Saldana, S., & Hayden, K. M. (2020). The association between neighborhood socioeconomic status, cardiovascular and cerebrovascular risk factors, and cognitive decline in the Health and Retirement Study (HRS). *Aging & Mental Health*, *24*(9), 1479–1486. https://doi.org/10.1080/13607863.2019.1594169 | - Not explicitly test life course models  - Not from a new country and dataset already included |
| 19 | Quiñones, A. R., Chen, S., Nagel, C. L., Botoseneanu, A., Allore, H. G., Newsom, J. T., Thielke, S., & Kaye, J. (2022). Trajectories of cognitive functioning in later life: Disparities by race/ethnicity, educational attainment, sex, and multimorbidity combinations. *SSM-Population Health*, *18*, 101084. https://doi.org/10.1016/j.ssmph.2022.101084 | Not explicitly test life course models |
| 20 | Kamalyan, L., Guarena, L. A., Díaz-Santos, M., Suarez, P., Cherner, M., Alcorn, M. Y. G., Umlauf, A., Franklin, D. R., Mindt, M. R., Fortuny, L. A., Heaton, R. K., & Marquine, M. J. (2022). Influence of educational background, childhood socioeconomic environment, and language use on cognition among Spanish-speaking Latinos living near the US-Mexico border. *Journal of the International Neuropsychological Society*, *28*(8), 876–890. https://doi.org/10.1017/s1355617721001028 | No measure of cognition in the “second half of life” |
| 21 | Han, F., Luo, C., Lv, D., Tian, L., & Qu, C. (2022). Risk factors affecting cognitive impairment of the elderly aged 65 and over: A cross-sectional study. *Frontiers in Aging Neuroscience*, *14*, 903794. https://doi.org/10.3389/fnagi.2022.903794 | Not explicitly test life course models |
| 22 | Zhang, K., & Zhang, W. (2021). Adverse childhood experiences and mild cognitive impairment in later life: Exploring rural/urban and gender differences using CHARLS. *Journal of Applied Gerontology*, *41*(5), 1454–1464. https://doi.org/10.1177/07334648211064796 | Not from a new country |
| 23 | Tsang, R. S. M., Gallacher, J. E., & Bauermeister, S. (2022). The long arm of childhood socioeconomic deprivation on mid- to later-life cognitive trajectories: A cross-cohort analysis. *Alzheimer's & Dementia: Diagnosis, Assessment & Disease Monitoring*, *14*(1), e12322. https://doi.org/10.1002/dad2.12322 | Not from a new country |
| 24 | Schrempft, S., Trofimova, O., Künzi, M., Draganski, B., Kliegel, M., & Stringhini, S. (2022). Life-course socioeconomic conditions and cognitive performance in older adults: A cross-cohort comparison. *Aging & Mental Health*, *27*(4), 745–754. https://doi.org/10.1080/13607863.2022.2084511 | Not from a new country |
| 25 | Muhammad, T., Sekher, T., & Srivastava, S. (2022). Association of objective and subjective socioeconomic markers with cognitive impairment among older adults: Cross-sectional evidence from a developing country. *BMJ Open*, *12*(8), e052501. https://doi.org/10.1136/bmjopen-2021-052501 | Not explicitly test life course models |
| 26 | Wong, Y. P., & Yang, H. (2022). The influence of subjective socioeconomic status on executive functions in middle-aged and older adults. *Aging, Neuropsychology, and Cognition*, *30*(4), 505–522. https://doi.org/10.1080/13825585.2022.2055738 | Not use a new database |
| 27 | Horvat, P., Richards, M., Malyutina, S., Pajak, A., Kubinova, R., Tamosiunas, A., Pikhart, H., Peasey, A., Marmot, M.G., & Bobak, M. (2014). Life course socioeconomic position and mid-late life cognitive function in Eastern Europe. *The Journals of Gerontology, Series B: Psychological Sciences and Social Sciences*, *69*(3), 470–481. https://doi.org/10.1093/geronb/gbu014 | Not explicitly test life course models |
| 28 | Peterson, R. L., Carvajal, S. C., McGuire, L. C., Fain, M. J., & Bell, M. L. (2019). State inequality, socioeconomic position and subjective cognitive decline in the United States. *SSM-Population Health*, *7*, 100357. https://doi.org/10.1016/j.ssmph.2019.100357 | Measured cognition subjectively |
| 29 | Migeot, J., Calivar, M., Granchetti, H., Ibáñez, A., & Fittipaldi, S. (2022). Socioeconomic status impacts cognitive and socioemotional processes in healthy ageing. *Scientific Reports*, *12*(1), 6048. https://doi.org/10.1038/s41598-022-09580-4 | Not use new SEP indicator |
| 30 | Racine Maurice, S., Hébert, A., Turcotte, V., Potvin, O., Hudon, C., & Duchesne, S. (2021). Childhood socioeconomic status does not predict late-life cognitive decline in the 1936 Lothian Birth Cohort. *Frontiers in Psychology*, *12*, 679044. https://doi.org/10.3389/fpsyg.2021.679044 | Not explicitly test life course models |
| 31 | Zhang, T., Lu, B., & Wang, X. (2022). Urban-rural disparity in cognitive performance among older Chinese adults: Explaining the changes from 2008 to 2018. *Frontiers in Public Health*, *10*, 843608. https://doi.org/10.3389/fpubh.2022.843608 | No focus on SEP indicators |
| 32 | Chiao, C., Botticello, A., & Fuh, J. L. (2014). Life-course socio-economic disadvantage and late-life cognitive functioning in Taiwan: Results from a national cohort study. *International Health*, *6*(4), 322–330. https://doi.org/10.1093/inthealth/ihu046 | Not from a new country |
| 33 | Doblhammer, G., van den Berg, G. J., & Fritze, T. (2013). Economic conditions at the time of birth and cognitive abilities late in life: Evidence from ten European countries. *PLOS ONE*, *8*(9), e74915. https://doi.org/10.1371/journal.pone.0074915 | Not from a new country |
| 34 | Kobayashi, L. C., Glymour, M. M., Kahn, K., Payne, C. F., Wagner, R. G., Montana, L., Mateen, F. J., Tollman, S. M., & Berkman, L. F. (2017). Childhood deprivation and later-life cognitive function in a population-based study of older rural South Africans. *Social Science & Medicine*, *190*, 20–28. https://doi.org/10.1016/j.socscimed.2017.08.009 | Not explicitly test life course models |
| 35 | Araújo, L. F., Giatti, L., Chor, D., Passos, V. M. A., & Barreto, S. M. (2014). Maternal education, anthropometric markers of malnutrition and cognitive function (ELSA-Brasil). *BMC Public Health*, *14*, 673. https://doi.org/10.1186/1471-2458-14-673 | Not explicitly test life course models |
| 36 | Bertola, L., Benseñor, I. M., Barreto, S. M., Giatti, L., Moreno, A. B., Viana, M. C., Lotufo, P. A., & Suemoto, C. K. (2021). Early life socioeconomic status predicts cognition regardless of education level. *European Journal of Neurology*, *28*(12), 3972–3978. https://doi.org/10.1111/ene.15042 | Not use a new database |
| 37 | Leist, A. K., Bar-Haim, E., & Chauvel, L. (2021). Inequality of educational opportunity at time of schooling predicts cognitive functioning in later adulthood. *SSM Population Health*,*15*, 100837. https://doi.org/10.1016/j.ssmph.2021.100837 | Not use a new database |
| 38 | Lee, A. R., Torres, J., & Flores Romero, K. R. (2024). Adult child financial disadvantage and the cognitive trajectories among older parents in the United States. *The Journals of Gerontology, Series B: Psychological Sciences and Social Sciences*, *79*(9), gbae123. https://doi.org/10.1093/geronb/gbae123 | Not use a new database  No life course SEP |
| 39 | Li, Y., Tang, Y., Lu, J., Wu, H., & Ren, L. (2024). The dilution effect of healthy lifestyles on the risk of cognitive function attributed to socioeconomic status among Chinese older adults: A national wide prospective cohort study. *Journal of Global Health*,*14*, 04010. https://doi.org/10.7189/jogh-14-04010 | Not use a new database  No life course SEP |

**Table S2. Summary of socioeconomic position indicators used in the studies, by life course SEP periods (n=42)**

| **First author,**  **year** | **SEP indicators** | | | | | |
| --- | --- | --- | --- | --- | --- | --- |
|  | **Childhood/adolescence** | **Education** | **Young adulthood** | **Middle age** | **Older age** | **Cumulative SEP/SEP mobility** |
| Everson-Rose,  2003 | parental education, paternal occupation, family financial status | total number of years of education | / | / | / | / |
| Luo,  2005 | parental education, paternal occupation, family financial status | total number of years of education | / | / | household income | cumulative SEP (dichotomized) and SEP mobility (stable low/downward/mixed/upward/stable high) of childhood SEP, education, and household income |
| Wilson,  2005 | parental education, paternal principal lifetime occupation, family financial status, *county socioeconomic index at birth* | total number of years of education | / | principal lifetime occupation | / | / |
| Zhang,  2008 | *born in urban region*, *went to bed without hunger* | total number of years of education | / | occupation, urban residence | / | / |
| Brown,  2010 | parental education, family SEP, paternal occupation | whether total number of years of education > 12 years | / | / | household income | / |
| Jefferson,  2011 | parental education, paternal occupation, number of children in the family, *community-level socioeconomic status* | total number of years of education | / | household income at age of 40 | occupation, household income | / |
| Barnes,  2012 | frequency of stories and games, family financial status, *food deprivation*, *self-rated health and body size* | total number of years of education | / | / | / | / |
| González,  2013 | parental education, socioeconomic status, *health status* | total number of years of education | / | / | household income, wealth | / |
| Hurst,  2013 | paternal occupation | highest educational achievement | / | occupation | / | / |
| Brewster,  2014 | parental education, paternal occupation, number of siblings, number of siblings who died during childhood | total number of years of education | / | / | / | / |
| Fritze,  2014 | urban/rural residence, living arrangement at age 10, occupation of main breadwinner at age 10, number of books in the household at age 10, financial hardship between birth and age 18, *economic conditions at time of birth (recession, average, boom)* | highest educational achievement | / | / | / | / |
| Leist,  2014 | *whether born before the World War II in 1945*, country of residence, childhood conditions at age 10, *number of recessions from birth up to age 49* | highest educational achievement | *first occupation* | / | / | life-course occupational class mobility and working conditions |
| Chen,  2016 | paternal education, *economic conditions during the year of birth and the year prior to birth (recession, boom)* | highest educational achievement | / | / | / | / |
| Lyu,  2016 | parental education, paternal occupation, family financial status | total number of years of education | / | / | annual household income | cumulative SEP (index) and *SEP mobility* (low stable/downward/upward/high stable) of childhood SEP, education, and household income |
| Marden,  2016 | parental education, paternal occupation, financial capital | high-school (≥ 12 years) or college (≥ 16 years) completion | / | / | household income, wealth, labour force status | Life course SEP trajectories (dichotomized childhood SEP, education, and older-age SEP) |
| Ritchie,  2016 | paternal occupation | total number of years of education | / | most prestigious occupation | Scottish Index of Multiple Deprivation | / |
| Staff,  2016 | paternal occupation | whether total number of years of education > 11 years | / | best occupational level | / | / |
| Ericsson,  2017 | parental occupation | highest educational achievement | / | / | / | / |
| Landy,  2017 | paternal occupation | highest educational achievement | / | occupation at age 36 or 43, civil service grade | / | / |
| Cermakova,  2018 | overcrowding, number of books in the home | total number of years of education | / | / | current employment | / |
| Zaninotto,  2018 | paternal occupation | highest educational achievement | / | / | wealth | / |
| Aartsen,  2019 | main breadwinner’s occupation, number of books at home, overcrowding, housing quality | highest educational achievement | / | main occupational position | satisfaction with household income | / |
| Cheval,  2019 | number of books at home, overcrowding, main breadwinner’s occupation, housing quality | highest educational achievement | / | main occupational position | satisfaction with household income | / |
| Moorman,  2019 | parental education, paternal occupation, family income | total number of years of education; academic performance; school context | / | occupational prestige, and household income at age 53 | / | / |
| Greenfield,  2020 | parental education, paternal occupation, household income | highest educational achievement, total number of years of education | / | household income, occupation | / | / |
| Maharani,  2020 | *hunger experience*, availability of facilities in the home at the age 12, number of books at home | highest educational achievement | / | / | employment status, household expenditure | / |
| Wahrendorf,  2020 | number of books at home, housing quality | highest educational achievement | / | adverse employment histories | employment situation | / |
| Yang,  2020 | paternal education, paternal occupation, *food adequacy* | highest educational achievement | / | / | / | / |
| Ding,  2021 | parental illiteracy, *hunger experience*, *flight away from famine*, worse economic situation than neighbours | total number of years of education | / | / | annual household expenditure per capita | / |
| Faul,  2021 | paternal/parental unemployment, financial difficulty before age 16, paternal occupation | whether total number of years of education > 12 years | / | / | household wealth | / |
| Kunzi,  2021 | paternal occupation, parental education | highest educational achievement | *first occupation* | last occupation | monthly income, household fortune, making ends meet or not | / |
| Lee,  2021 | *family life happiness*, family structure, parental education, family financial status | highest educational achievement | / | *social connectedness*, *self-mastery*, *self-rated health*, *adulthood resources* | / | / |
| Leist,  2021 | number of books at home, *relative school performance in mathematics and language at age 10* | highest educational achievement; inequality of educational opportunity | / | / | current job situation | / |
| Peterson,  2021 | financial capital, cultural capital, social capital | whether highest educational achievement < high school/passing the general educational development test | / | / | financial capital, cultural capital, social capital | SEP trajectories (consistently high capital, high childhood and low older-age capital, high older-age and low childhood capital, consistently low capital) |
| Selvamani,  2021 | parental education, parental employment status | highest educational achievement | / | employment status | wealth | education-based life course SES trajectories, employment-based life course SES trajectories |
| Wolfova,  2021 | overcrowding, number of books at home | total number of years of education | / | / | household net worth, current working status | / |
| Ford,  2022 | lower-skilled occupation of the breadwinner, having few books at home, overcrowding, no indoor toilet, no fixed bath, no central heating, and no hot and cold running water supply to the home | highest educational achievement | / | occupational levels | / | / |
| Muhammad,  2022 | family financial status | highest educational achievement | / | / | social participation | / |
| Reynolds,  2022 | human capital, social capital, financial capital, *region of residence (south vs. non-south)* | whether total number of years of education > 16 years | / | / | / | */* |
| Ye,  2022 | family financial status, parental education | total number of years of education | / | / | household monthly consumption per capita | */* |
| Zeng,  2022 | parental education, paternal occupation, family financial status | highest educational achievement | *occupation (whether white-collar)* | household income, net worth | / | cumulative SEP disadvantages (high/moderate/low/no exposure), SEP mobility (stable high/downward /upward/stable low) |
| Zhou,  2022 | parental education, parental employment, whether experiencing starvation, family financial status | highest educational achievement | / | / | household expenditure, employment | / |

*Note*.

SEP = socioeconomic position. / = not included*.* Text in *Italic* shows results of SEP indicators that are unique and not used by other studies.

**Table S3. Summary of associations between individual-level SEP indicators and cognitive levels and trajectories in various domains, by life course SEP periods (n=16)**

| **Life course periods** | **Dimensions of SEP^1^** | **Memory** | | **Executive functioning** | | **Processing speed** | | **Sensation, perception** | | **Motor skills** | | **Language, verbal skills** | |
| --- | --- | --- | --- | --- | --- | --- | --- | --- | --- | --- | --- | --- | --- |
|  |  | **Levels** | **Trajectories** | **Levels** | **Trajectories** | **Levels** | **Trajectories** | **Levels** | **Trajectories** | **Levels** | **Trajectories** | **Levels** | **Trajectories** |
| **Childhood/adolescence** | Education | + (12) | + (2)  - (1)  × (3) | + (4) |  | + (3)  × (1) | × (1) | + (2) | × (1) | + (1) |  | + (5) | + (2)  × (2) |
|  | Income | + (2) |  | + (2) |  |  |  |  |  |  |  |  |  |
|  | Occupation | + (8)  × (1) | + (1)  × (2) | + (4) |  | + (3) | + (1)  × (1) | + (1) |  |  |  | + (3) | × (2) |
|  | Wealth |  |  |  |  |  |  |  |  |  |  |  |  |
| **Young adulthood** | Education | + (7)  × (1) | × (1) | + (4) |  | + (2)  × (1) | × (1) | + (1) |  | + (1) |  | × (1) | × (1) |
|  | Income |  |  |  |  |  |  |  |  |  |  |  |  |
|  | Occupation |  |  |  |  |  |  |  |  |  |  |  |  |
|  | Wealth |  |  |  |  |  |  |  |  |  |  |  |  |
| **Middle age** | Education |  |  |  |  |  |  |  |  |  |  |  |  |
|  | Income | × (1) |  |  |  | + (1) |  | × (1) |  |  |  |  |  |
|  | Occupation | + (4) | + (2)  × (1) |  |  | + (1)  × (1) | × (1) |  |  | + (1) |  | + (3)  × (1) | + (1)  × (2) |
|  | Wealth |  |  |  |  |  |  |  |  |  |  |  |  |
| **Older age** | Education |  |  |  |  |  |  |  |  |  |  |  |  |
|  | Income | + (3)  × (1) |  | + (1) |  | + (1) |  | × (1) |  |  |  |  |  |
|  | Occupation | + (3)  × (1) |  | + (1) |  | + (1) |  | × (1) |  |  |  |  |  |
|  | Wealth | + (2)  × (1) | × (1) | + (1) | × (1) | + (1) | × (1) |  |  |  |  |  |  |

SEP= socioeconomic position. Each SEP indicator reflects the parents’ characteristics when the life course period is childhood and the individuals for the other periods.

^1^ Dimensions of individual-level SEP refer to the “big four” model (Hällsten & Thaning, 2022).

^2^ Symbols for effect direction: + protective, - risk, × null (not statistically significant). Each cell counts the studies which showed the abovementioned effect direction.

**Preferred Reporting Items for Systematic reviews and Meta-Analyses extension for Scoping Reviews (PRISMA-ScR) Checklist**

| **SECTION** | **ITEM** | **PRISMA-ScR CHECKLIST ITEM** | **REPORTED ON PAGE #** |
| --- | --- | --- | --- |
| **TITLE** | | | |
| Title | 1 | Identify the report as a scoping review. | 1 |
| **ABSTRACT** | | | |
| Structured summary | 2 | Provide a structured summary that includes (as applicable): background, objectives, eligibility criteria, sources of evidence, charting methods, results, and conclusions that relate to the review questions and objectives. | 2-3 |
| **INTRODUCTION** | | | |
| Rationale | 3 | Describe the rationale for the review in the context of what is already known. Explain why the review questions/objectives lend themselves to a scoping review approach. | 4 |
| Objectives | 4 | Provide an explicit statement of the questions and objectives being addressed with reference to their key elements (e.g., population or participants, concepts, and context) or other relevant key elements used to conceptualize the review questions and/or objectives. | 5 |
| **METHODS** | | | |
| Protocol and registration | 5 | Indicate whether a review protocol exists; state if and where it can be accessed (e.g., a Web address); and if available, provide registration information, including the registration number. | 2 |
| Eligibility criteria | 6 | Specify characteristics of the sources of evidence used as eligibility criteria (e.g., years considered, language, and publication status), and provide a rationale. | 9-10 |
| Information sources* | 7 | Describe all information sources in the search (e.g., databases with dates of coverage and contact with authors to identify additional sources), as well as the date the most recent search was executed. | 8-9 |
| Search | 8 | Present the full electronic search strategy for at least 1 database, including any limits used, such that it could be repeated. | 8-9 |
| Selection of sources of evidence† | 9 | State the process for selecting sources of evidence (i.e., screening and eligibility) included in the scoping review. | 8-9 |
| Data charting process‡ | 10 | Describe the methods of charting data from the included sources of evidence (e.g., calibrated forms or forms that have been tested by the team before their use, and whether data charting was done independently or in duplicate) and any processes for obtaining and confirming data from investigators. | 8-9 |
| Data items | 11 | List and define all variables for which data were sought and any assumptions and simplifications made. | 8-9 |
| Critical appraisal of individual sources of evidence§ | 12 | If done, provide a rationale for conducting a critical appraisal of included sources of evidence; describe the methods used and how this information was used in any data synthesis (if appropriate). | / |
| Synthesis of results | 13 | Describe the methods of handling and summarizing the data that were charted. | 10-12 |
| **RESULTS** | | | |
| Selection of sources of evidence | 14 | Give numbers of sources of evidence screened, assessed for eligibility, and included in the review, with reasons for exclusions at each stage, ideally using a flow diagram. | 13 |
| Characteristics of sources of evidence | 15 | For each source of evidence, present characteristics for which data were charted and provide the citations. | 13-16 |
| Critical appraisal within sources of evidence | 16 | If done, present data on critical appraisal of included sources of evidence (see item 12). | / |
| Results of individual sources of evidence | 17 | For each included source of evidence, present the relevant data that were charted that relate to the review questions and objectives. | 16-22 |
| Synthesis of results | 18 | Summarize and/or present the charting results as they relate to the review questions and objectives. | 16-25 |
| **DISCUSSION** | | | |
| Summary of evidence | 19 | Summarize the main results (including an overview of concepts, themes, and types of evidence available), link to the review questions and objectives, and consider the relevance to key groups. | 26-35 |
| Limitations | 20 | Discuss the limitations of the scoping review process. | 36-40 |
| Conclusions | 21 | Provide a general interpretation of the results with respect to the review questions and objectives, as well as potential implications and/or next steps. | 40 |
| **FUNDING** | | | |
| Funding | 22 | Describe sources of funding for the included sources of evidence, as well as sources of funding for the scoping review. Describe the role of the funders of the scoping review. | 2 |

JBI = Joanna Briggs Institute; PRISMA-ScR = Preferred Reporting Items for Systematic reviews and Meta-Analyses extension for Scoping Reviews.

* Where *sources of evidence* (see second footnote) are compiled from, such as bibliographic databases, social media platforms, and Web sites.

† A more inclusive/heterogeneous term used to account for the different types of evidence or data sources (e.g., quantitative and/or qualitative research, expert opinion, and policy documents) that may be eligible in a scoping review as opposed to only studies. This is not to be confused with *information sources* (see first footnote).

‡ The frameworks by Arksey and O’Malley (6) and Levac and colleagues (7) and the JBI guidance (4, 5) refer to the process of data extraction in a scoping review as data charting*.*

§ The process of systematically examining research evidence to assess its validity, results, and relevance before using it to inform a decision. This term is used for items 12 and 19 instead of "risk of bias" (which is more applicable to systematic reviews of interventions) to include and acknowledge the various sources of evidence that may be used in a scoping review (e.g., quantitative and/or qualitative research, expert opinion, and policy document).

*From:* Tricco AC, Lillie E, Zarin W, O'Brien KK, Colquhoun H, Levac D, et al. PRISMA Extension for Scoping Reviews (PRISMAScR): Checklist and Explanation. Ann Intern Med. 2018;169:467–473. [doi: 10.7326/M18-0850](http://annals.org/aim/fullarticle/2700389/prisma-extension-scoping-reviews-prisma-scr-checklist-explanation).
